# Diagnostic accuracy of tongue diagnosis for stroke syndrome differentiation in traditional Chinese medicine: a systematic review, meta-analysis, and narrative synthesis of artificial intelligence-assisted models

**DOI:** 10.3389/fneur.2026.1874937

**Published:** 2026-10-07

**Authors:** Yibo Huang, Jiashun Yin, Siyuan Shao, Pengyu Jiang, Zilong Wang, Hui Xu

**Affiliations:** 1 School of Acupuncture, Moxibustion and Tuina, Anhui University of Chinese Medicine, Hefei, Anhui, China; 2 School of Medical Information Engineering, Anhui University of Chinese Medicine, Hefei, Anhui, China; 3 The Second Affiliated Hospital of Anhui University of Chinese Medicine, Hefei, Anhui, China; 4 The First Affiliated Hospital of Anhui University of Chinese Medicine, Hefei, Anhui, China; 5 Department of Clinical Medicine, North Anhui Health Vocational College, Suzhou, Anhui, China; 6 First Clinical Medical College, Anhui University of Chinese Medicine, Hefei, Anhui, China

**Keywords:** artificial intelligence, diagnostic test accuracy, GRADE-DTA, meta-analysis, stroke, syndrome differentiation, tongue diagnosis, traditional Chinese medicine

## Abstract

**Background:**

Tongue examination is central to traditional Chinese medicine (TCM) syndrome differentiation after stroke, but its diagnostic accuracy has not been evaluated using contemporary diagnostic test accuracy (DTA) methods.

**Methods:**

We searched PubMed, Embase, Web of Science, CNKI, Wanfang, and VIP from inception to 23 February 2026 (PROSPERO: CRD420261288502). Track 1 studies of manual tongue diagnosis were pooled using bivariate or univariate random-effects models; Track 2 studies of artificial intelligence (AI)-assisted models were synthesized narratively according to SWiM. Risk of bias was assessed with QUADAS-2 and the certainty of Track 1 evidence with GRADE-DTA.

**Results:**

Ten studies from mainland China included 3,437 participants: six manual-diagnosis studies (*N* = 2,191) and four AI-assisted studies (*N* = 1,246). Because evidence certainty was very low and reference-standard independence was not assured, all estimates were treated as exploratory agreement with existing syndrome-classification processes rather than clinically validated diagnostic accuracy. The Deficient-Cold Tongue cluster showed pooled specificity of 0.97 (95% CI: 0.87–1.00) and a positive likelihood ratio (PLR) of 31.0 (95% CI: 2.8–341.6). The Greasy Coating cluster showed pooled sensitivity of 0.83 (95% CI: 0.52–0.96), specificity of 0.80 (95% CI: 0.68–0.88), PLR of 4.1 (95% CI: 2.5–6.8), and a negative likelihood ratio of 0.21 (95% CI: 0.06–0.71). Blood Stasis Tongue showed low sensitivity and unstable, heterogeneous estimates. The small analyzable evidence base contrasted with the much larger descriptive literature.

**Conclusion:**

Current evidence remains too uncertain to support manual tongue diagnosis for routine or standalone clinical use, and applicability is largely limited to mainland Chinese TCM stroke-care settings. Manual and AI-assisted approaches require prospective multicenter validation using independent syndrome adjudication that excludes the index tongue feature. Future studies should stratify analyses by stroke etiology and report calibration and clinical utility for AI models.

**Systematic review registration:**

PROSPERO, CRD420261288502.

## 1 Introduction

Stroke is the second leading cause of death and the third leading cause of long-term disability worldwide, accounting for approximately 11.6 million new cases and 5.5 million deaths annually (1). Between 1990 and 2019, the absolute number of stroke cases increased by 70%, with the greatest increase occurring in low and middle-income countries in East and Southeast Asia (1, 2). In parts of these regions, traditional Chinese medicine (TCM) syndrome differentiation is routinely integrated with neuroimaging to guide individualized herbal prescriptions, acupuncture, and rehabilitation (3). A recent review described the integration of TCM herbal medicine and acupuncture with conventional stroke management (4). Among the four diagnostic pillars of TCM, tongue examination (she zhen) is particularly valued because it requires no equipment, incurs no cost, and can be repeated at the bedside within seconds (5). Given its widespread role in treatment selection, the diagnostic contribution of tongue manifestations must be quantified before they can be used as evidence-informed adjuncts in stroke care. However, despite decades of observational research, tongue manifestations for stroke syndrome differentiation have not been evaluated using contemporary diagnostic test accuracy (DTA) standards.

Two methodological limitations have impeded such evaluation. First, most studies are descriptive, reporting the frequency of tongue features across syndromes without the 2 × 2 contingency tables needed to estimate sensitivity, specificity, and likelihood ratios (6). Second, TCM reference standards—typically national consensus guidelines—are composite criteria that include tongue signs as diagnostic elements (5). This overlap creates incorporation bias, a structural problem affecting the evidence base rather than any single study (7). A further concern is that tongue diagnosis relies on unaided visual assessment by individual practitioners, yet interobserver agreement in stroke populations has not been systematically quantified (8).

During the past decade, convolutional neural networks, including ResNet and DenseNet, have been applied to automated tongue-image classification, and several single-center studies have reported internal accuracies above 90% (9–11). These models may support more consistent and reproducible image interpretation, but their performance depends on model architecture, image-acquisition conditions, labeling quality, and validation design. The evidence remains fragmented: existing studies have generally used artificially balanced, single-center datasets and have lacked independent external validation. Moreover, no systematic review has appraised these AI-assisted models against established reporting standards for AI-assisted diagnostic and prediction models (12, 13).

We therefore conducted a prospectively registered systematic review and meta-analysis using a dual-track approach. Track 1 quantitatively synthesized studies of manual visual tongue diagnosis using bivariate (14) and univariate (15) random-effects DTA models. Track 2 narratively synthesized AI-assisted tongue-recognition models that could not be pooled, following the Synthesis Without Meta-analysis (SWiM) framework (16). We assessed risk of bias with QUADAS-2 (17) and rated the certainty of Track 1 evidence with GRADE-DTA (18). To our knowledge, this is the first systematic review to apply formal DTA methods to manual tongue diagnosis for stroke syndrome differentiation while critically appraising emerging AI-assisted approaches. Because current TCM reference standards may incorporate the tongue features under evaluation, our aim was to summarize apparent diagnostic performance under existing classification processes and to determine whether current study designs support clinically valid accuracy estimates.

## 2 Materials and methods

### 2.1 Protocol and registration

This systematic review was prospectively registered in PROSPERO (CRD420261288502) and conducted according to the PRISMA 2020 (19) and PRISMA-DTA (20) guidelines. The completed checklist was provided in Supplementary File S3. One amendment was made after registration. During full-text screening, we identified two reports from the same research group, same institution, and overlapping enrollment period (Cao journal article 2021; Cao master’s thesis 2021). To avoid unit-of-analysis error, we retained only the more comprehensive report (master’s thesis, *n* = 150) and excluded the journal article (*n* = 151), following Cochrane recommendations on overlapping cohorts (21). This change was documented before data extraction.

### 2.2 Eligibility criteria

We used the PIRTS framework for diagnostic accuracy reviews (6). The population included patients with stroke (ischemic or hemorrhagic) confirmed by CT or MRI. The index test was either traditional manual visual tongue assessment by qualified TCM practitioners (Track 1) or artificial intelligence-assisted quantitative tongue recognition (Track 2). The reference standard consisted of nationally endorsed TCM consensus guidelines for stroke syndrome classification. Target conditions were pre-specified TCM pathomechanisms (Qi-deficiency and blood-stasis, wind-phlegm and dampness, phlegm-heat and excess fire, yin-deficiency). Cross-sectional, prospective, and retrospective cohort studies were eligible. Syndrome-vs-other-syndrome case–control designs were eligible for Track 1 when controls were other stroke syndrome groups rather than healthy participants. Healthy-control AI case–control studies were retained only for narrative exploration in Track 2 and were downgraded for bias and applicability because they did not directly answer the clinical question of syndrome differentiation among patients with stroke. Reports were excluded if they did not enroll patients with neuroimaging-confirmed stroke, did not evaluate tongue manifestations or tongue images as the index test, did not use nationally endorsed TCM syndrome criteria as the reference standard, or did not provide sufficient diagnostic information for the intended quantitative or narrative synthesis.

### 2.3 Search strategy

We searched PubMed, Embase, Web of Science, CNKI, Wanfang, and VIP from inception to 23 February 2026 using a combination of subject headings and free-text terms for stroke and tongue diagnosis, with no language restriction. The full search strategies were provided in Supplementary Appendix S1. We also hand-searched reference lists of included studies and relevant reviews.

### 2.4 Study selection and data extraction

Duplicate records were identified and removed using Zotero (version 7.0; Corporation for Digital Scholarship) and manual checking. In total, 451 duplicate records were removed before screening: 370 by automated duplicate detection and 81 by manual confirmation. No automation tool was used to exclude records for ineligible content before manual title/abstract screening. Two reviewers independently screened titles, abstracts, and full texts; all eligibility decisions were made manually. Disagreements were resolved by consensus or adjudication by a third senior reviewer.

Data were independently extracted by two reviewers using a piloted form, and extraction discrepancies were resolved using the same consensus/adjudication process. For each tongue feature within each diagnostic cluster, we extracted the 2 × 2 contingency table (true positives, false positives, false negatives, true negatives). When studies reported sensitivity, specificity, and sample size without a contingency table, we performed algebraic back-calculation and rounded fractional cell counts to the nearest integer to satisfy binomial distribution requirements. We additionally classified each 2 × 2 row by provenance as directly reported, back-calculated from sensitivity/specificity, derived from overlapping-label arithmetic, or extracted from direct two-group counts; denominator checks, back-calculation procedures, and rounding residuals were documented in Supplementary Table S9. We recorded study design, enrollment period, country or region, healthcare setting when reported, stroke subtype and stage, sample size, demographics, assessor qualifications, image-acquisition context for AI studies when available, and the exact reference standard used. Items not reported were coded as “NR.”

### 2.5 Risk of bias assessment

Two reviewers independently applied QUADAS-2 (17) across the four domains (patient selection, index test, reference standard, flow and timing). We defined an ideal test accuracy study as a prospective, fully blinded design in which an independent committee establishes the syndrome diagnosis using comprehensive clinical and neuroimaging data; this benchmark guided severity grading, particularly for incorporation bias in the reference standard domain. For artificial intelligence studies (Track 2), we additionally evaluated data leakage, post-hoc threshold optimization, absence of independent external validation, and spectrum bias from image preprocessing, using core items from STARD-AI (13) and TRIPOD+AI (12). Disagreements were resolved by discussion with the third reviewer.

### 2.6 Statistical analysis

Clinical feature clustering. We mapped heterogeneous tongue features to eight pre-specified diagnostic clusters organized under four major TCM pathomechanisms. A hierarchical rule (tongue body morphology > coating characteristics > sublingual vasculature) prevented double-counting within clusters.

Data synthesis. We used a stratified approach based on available degrees of freedom. The unit of assessment for Track 1 diagnostic accuracy estimates was the participant-level 2 × 2 table for each tongue-feature cluster whenever such data were directly reported or reconstructable. Track 2 artificial intelligence studies were not pooled because standardized participant-level 2 × 2 data were not available and some studies reported image-level rather than patient-level validation structures. For the Greasy Coating cluster (*k* = 4), we applied a bivariate random-effects model (14) to jointly estimate sensitivity and specificity and generate a summary SROC curve (22); Because this model was fitted with only four studies, its convergence status, log likelihood, random-effects covariance structure, and binomial GLMM sensitivity check were reported in Supplementary Table S10. The summary operating point was interpreted cautiously as an exploratory estimate. For the accompanying forest plots and for clusters with *k* = 3, we used univariate random-effects models with the DerSimonian-Laird estimator (15), Freeman-Tukey double-arcsine transformation, and exact binomial study-level confidence intervals (23). Because several proportions were close to the boundary, we performed logit-transformed random-effects sensitivity analyses for sensitivity and specificity and compared them with the Freeman-Tukey estimates in Supplementary Table S10. Forest plots therefore displayed descriptive univariate estimates and were not expected to be numerically identical to the Greasy Coating bivariate SROC summary point. For the two Yin-Deficiency clusters (*k* = 2) quantitative pooling was not performed per Cochrane DTA guidance (23); we presented the data as unpooled evidence maps only. For PLR, NLR, and DOR, study-level log-transformed likelihood ratios and diagnostic odds ratios were pooled using random-effects models; A 0.5 continuity correction was applied only to studies containing a zero cell and only for LR/DOR calculations. Greasy Coating LR and DOR values reported as the primary result were taken from the bivariate model summary point.

Heterogeneity and threshold effect. Heterogeneity was quantified using *I*^2^ (24). When the between-study variance estimator (τ^2^) converged, it was reported. In sparse networks (*k* ≤ 3) with boundary values, I^2^ was recorded as not applicable when variance estimation was unstable; substantial heterogeneity was instead inferred from the width of the confidence intervals. The pre-specified Spearman correlation between logit-transformed sensitivity and the logit-transformed false-positive rate could not be calculated because of extreme sparsity.

Sensitivity analysis. We performed leave-one-out sensitivity analyses for all quantitatively pooled clusters. Because excluding one study from a *k* = 4 cluster reduced the network to *k* = 3, all iterations used univariate models to maintain stability. We also performed three additional methodological sensitivity checks to address uncertainty arising from sparse model fitting, transformation choice, and reconstructed 2 × 2 data: Greasy Coating bivariate-model diagnostics with binomial GLMM comparison, Freeman-Tukey versus logit-transformed univariate sensitivity analyses, and affected-row exclusion analyses based on the 2 × 2 provenance audit in Supplementary Table S9. Rows with *k* = 2 after exclusion were reported only as descriptive influence checks and were not interpreted as confirmatory pooled estimates. These methodological sensitivity analyses were summarized in Supplementary Table S10. Illustrative post-test probability analysis. These analyses were intended to illustrate possible post-test probability changes and were not interpreted as evidence of clinical utility. We constructed Fagan nomograms (25) using pooled likelihood ratios.

A 50% pre-test probability was used as a standardized hypothetical reference rather than an assumed real-world syndrome prevalence, because syndrome prevalence varied substantially across studies and target syndromes; additional scenarios at 20 and 80% assessed robustness across prevalence ranges.

Reporting bias. Formal Deeks’ test (26) was not performed because all clusters contained fewer than ten studies.

Certainty of evidence. We evaluated certainty for Track 1 clusters using GRADE-DTA (18) in GRADEpro GDT (27). Two reviewers independently assessed risk of bias, indirectness, inconsistency, imprecision, and publication bias. Indirectness was considered when the evidence was restricted to a single geographic, cultural, clinical, or healthcare-system setting, or when the intended clinical or AI-deployment context differed from the populations and settings represented in the included studies. Absolute effects at a standardized 50% pre-test probability were reported in Table 1; supplementary analyses were cited alongside the corresponding results.

**Table 1:** GRADE-DTA summary of findings (SoF) for manual tongue diagnosis in stroke.

Index test cluster	k (N)	Pooled Se/Sp (95% CI)	Absolute effects per 1,000 (TP/FP/FN/TN)	PLR (95% CI)	NLR (95% CI)	Certainty
1. Wind-phlegm and dampness syndrome						
Greasy coating	4 (1068)	Se: 0.83 (0.52, 0.96) Sp: 0.80 (0.68, 0.88)	TP: 415 (260–480) FP: 100 (60–160) FN: 85 (20–240) TN: 400 (340–440)	4.1 (2.5, 6.8)	0.21 (0.06, 0.71)	⊕◯◯◯ Very low a,b,c,d
Phlegm-stasis tongue	3 (648)	Se: 0.78 (0.31, 1.00) Sp: 0.79 (0.56, 0.96)	TP: 390 (155–500) FP: 105 (20–220) FN: 110 (0–345) TN: 395 (280–480)	3.8 (1.2, 12.7)	0.20 (0.04, 1.02)	⊕◯◯◯ Very low a,b,c,d
2. Qi-deficiency and blood stasis syndrome						
Deficient-cold tongue	3 (1513)	Se: 0.74 (0.36, 0.99) Sp: 0.97 (0.87, 1.00)	TP: 370 (180–495) FP: 15 (0–65) FN: 130 (5–320) TN: 485 (435–500)	31.0 (2.8, 341.6)	0.24 (0.07, 0.88)	⊕◯◯◯ Very low a,b,c,d
Qi-Def morphology	3 (648)	Se: 0.88 (0.73, 0.98) Sp: 0.81 (0.38, 1.00)	TP: 440 (365–490) FP: 95 (0–310) FN: 60 (10–135) TN: 405 (190–500)	7.4 (0.76, 71.7)	0.24 (0.07, 0.85)	⊕◯◯◯ Very low a,b,c,d
Blood stasis tongue	3 (678)	Se: 0.24 (0.07, 0.46) Sp: 0.88 (0.60, 1.00)	TP: 120 (35–230) FP: 60 (0–200) FN: 380 (270–465) TN: 440 (300–500)	2.83 (0.45, 17.68)	0.88 (0.65, 1.19)	⊕◯◯◯ Very low a,b,c,d
3. Phlegm-heat and excess fire syndrome						
Heat syndrome tongue	3 (566)	Se: 0.74 (0.50, 0.93) Sp: 0.78 (0.55, 0.95)	TP: 370 (250–465) FP: 110 (25–225) FN: 130 (35–250) TN: 390 (275–475)	2.86 (1.76, 4.66)	0.42 (0.29, 0.59)	⊕◯◯◯ Very low a,b,c,d
4. Yin-deficiency syndrome (evidence mapping only—pooling not performed at *k* = 2)						
Yin-Def coating (peeled/scanty)	2 (478)	Se: 0.67–1.00 Sp: 0.92–0.99	Not applicable (pooling not performed)	N/A	N/A	⊕◯◯◯ Very low a,b,f,d
Yin-Def body (red/thin)	2 (478)	Se: 0.60–0.78 Sp: 1.00	Not applicable (pooling not performed)	N/A	N/A	⊕◯◯◯ Very low a,b,f,d

Because independence between index tongue features and composite TCM syndrome reference standards was not assured, the estimates in this table represent apparent or provisional agreement with current syndrome-classification processes rather than diagnostic accuracy against an independent reference standard. Sensitivity, specificity, likelihood ratios, and absolute effects may therefore be inflated to an unknown degree.

a. risk of bias: downgraded by 2 levels (Very Serious) across all clusters. Serious potential for incorporation bias at the body-of-evidence level: independence between evaluated tongue features and composite TCM syndrome reference standards was not assured across Track 1 studies, limiting interpretation in a way that cannot be remedied by meta-analytic adjustment.

b. indirectness: downgraded by 1 level (Serious) across all clusters. All included studies were conducted in mainland China and the evidence base was geographically, culturally, clinically, and healthcare-system specific; direct applicability to non-mainland Chinese, non-Asian, or Western integrative-medicine settings remains uncertain.

c. inconsistency: downgraded by 1–2 levels (Serious to Very Serious). Severe unexplained between-study heterogeneity (*I*^2^ > 80% in most clusters) is attributable to inter-observer variation in visual assessment thresholds in index-test interpretation. Formal threshold effect testing via Spearman’s rank correlation was mathematically precluded owing to extreme network sparsity (*k* ≤ 4 per cluster).

d. imprecision: downgraded by 1–2 levels (Serious to Very Serious). Extreme sparse evidence networks (*k* ≤ 4 per cluster) produced wide 95% confidence intervals, boundary-value instability (e.g., specificity upper bounds of 1.00), and non-convergent or unreliable between-study variance estimates.

e. publication bias: not formally assessed. Deeks’ funnel plot asymmetry test is statistically underpowered at *k* < 10 (Cochrane DTA Handbook, Chapter 10). Language bias favoring Chinese-language literature is acknowledged qualitatively but cannot be quantified with the available data.

f. inconsistency not assessable: For Yin-Deficiency clusters (*k* = 2), *I*^2^ and τ^2^ are mathematically incalculable at one degree of freedom. Quantitative pooling was not performed to prevent spurious precision. The inconsistency domain is recorded as not assessable, not rated up or down.

*Blood Stasis Tongue: Recalculated estimates indicate low sensitivity, high heterogeneity, and imprecise likelihood ratios (PLR 2.83, 95% CI 0.45–17.68; NLR 0.88, 95% CI 0.65–1.19), not a reliable inverse diagnostic association. This cluster should be interpreted as exploratory; future primary studies should stratify by stroke etiology and use reference standards that do not incorporate the index tongue feature.

Pre-test probability standardized at 50% (500 cases per 1,000 patients). Numbers in parentheses = 95% confidence intervals. Three-scenario data and heterogeneity statistics (τ^2^, *I*^2^) are provided in Supplementary Tables S8 and S6, respectively. All estimates are exploratory and should not be interpreted as clinically validated diagnostic performance because the certainty of evidence was very low across all clusters.

Qualitative synthesis of artificial intelligence studies. Track 2 studies lacked standardized 2 × 2 data and were synthesized narratively according to the SWiM guideline (16). For each AI-assisted study, we extracted, when reported, the model architecture, image-acquisition approach, preprocessing and augmentation procedures, validation strategy, number of participants and images, image-to-participant ratio, split level, patient-level separation, best reported performance, baseline or human-expert comparator, calibration reporting, decision-curve analysis, comparator type, and external-validation status. Reporting and model-development quality were appraised using core items from STARD-AI (13) and TRIPOD+AI (12), focusing on data leakage, post-hoc threshold optimization, external validation, calibration, human-comparator benchmarking, and spectrum bias. The selection of these appraisal domains was also consistent with recent AI-healthcare reporting-guidance discussions emphasizing transparent reporting of model development and evaluation and the distinction between internal and external testing (28). These reporting guidelines were used as appraisal frameworks rather than formal risk-of-bias instruments.

Primary Track 1 analyses were performed in Stata 19 (29). The 2 × 2 provenance, back-calculation, and rounding-residual audit was documented in Supplementary Table S9, whereas model-diagnostics, transformation-sensitivity, and affected-row exclusion analyses were documented in Supplementary Table S10.

## 3 Results

### 3.1 Study selection and characteristics

Searches across six electronic databases yielded 13,895 records. After removal of 451 duplicate records (370 identified automatically and 81 confirmed manually), 13,444 unique records were screened by title and abstract.

At this stage, 13,109 records were excluded for ineligible content (*n* = 9,833), animal research (*n* = 1,656), non-original publications (*n* = 1,110), inaccessible abstracts (*n* = 504), or retraction (*n* = 6). The remaining 335 records were retrieved in full text. Of these, 325 did not meet the eligibility criteria, most commonly because they were not diagnostic accuracy studies (*n* = 123), lacked sufficient data for contingency table construction (*n* = 66), used an incorrect reference standard (*n* = 57), had an unclear index test (*n* = 41), or were duplicate publications (*n* = 37). One record was excluded for overlapping patient cohort. Ten studies were included in the final synthesis (Figure 1).

**Figure 1:**
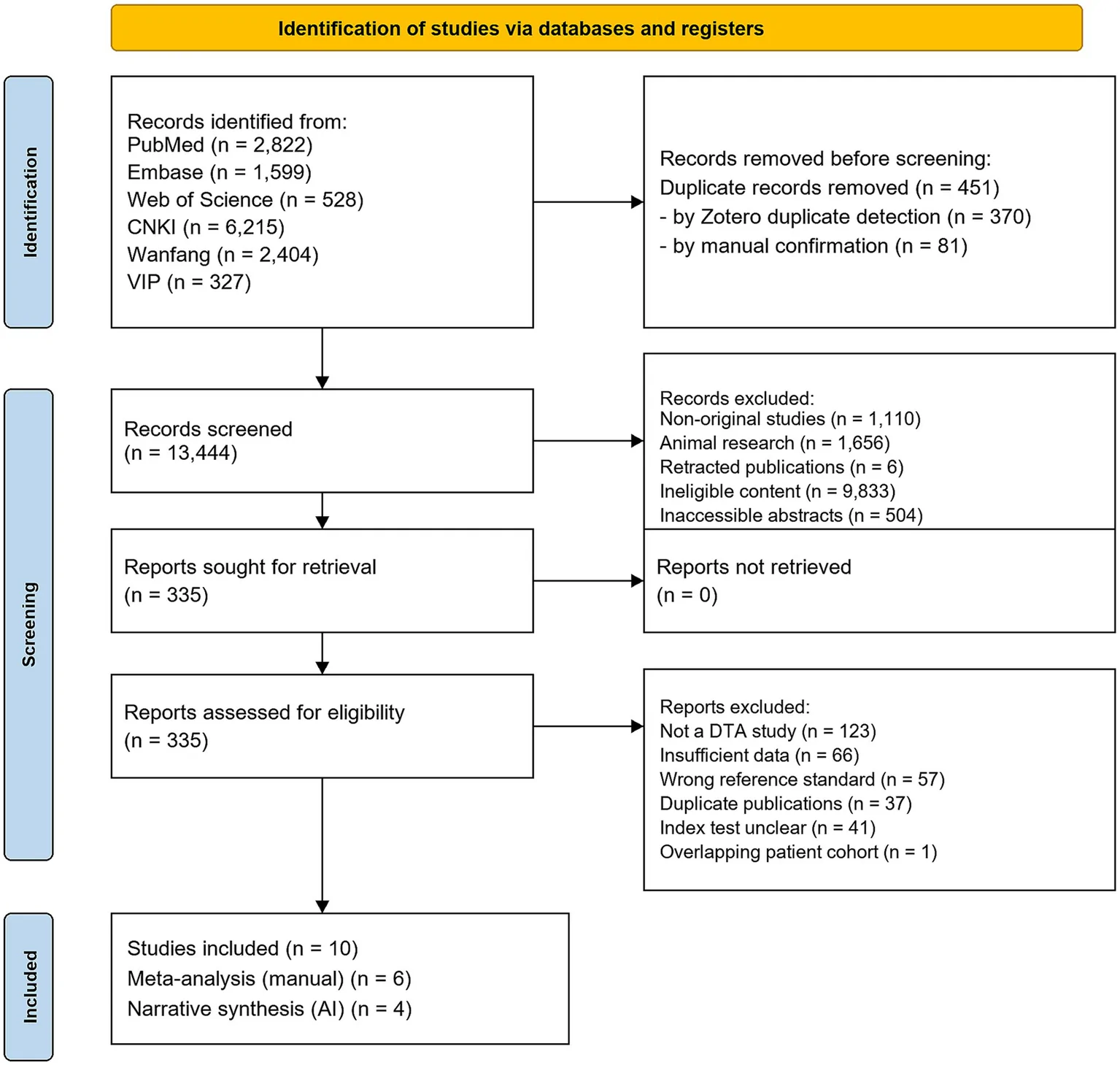
PRISMA 2020 flow diagram of study selection. A total of 13,895 records were identified from six electronic databases. After removing 451 duplicate records (370 identified by Zotero duplicate detection and 81 confirmed manually), 13,444 records were screened by title and abstract, 335 records were assessed at full text, and ten studies (3,437 participants) met the eligibility criteria and were included in the final dual-track synthesis.

These ten studies enrolled 3,437 participants. All ten included studies were conducted in mainland China, indicating a geographically concentrated evidence base. Sample sizes ranged from 88 to 1,035, and publication years spanned 2001 to 2025. Six studies used manual visual tongue diagnosis and contributed to quantitative meta-analysis (Track 1; *N* = 2,191) (30–35), whereas four used artificial intelligence-assisted systems and were synthesized narratively (Track 2; *N* = 1,246) (36–39). Stroke subtypes were predominantly ischemic (*k* = 6), with one hemorrhagic study and three mixed populations. The target conditions encompassed six TCM stroke syndromes: wind-phlegm-stasis obstruction, Qi-deficiency with blood stasis, phlegm-heat obstructing fu-organs, yin-deficiency with wind stirring, wind-fire disturbing upper, and Liver-Qi stagnation; pinyin transliterations were provided in Table 2, and full extraction fields and detailed Table 2 footnotes were provided in Supplementary Table S5.

**Table 2:** Concise characteristics of included studies.

Study	Track/design	Population (*N*)	Target condition	Index test	Reference standard/key note
Track 1: manual visual tongue diagnosis (quantitative synthesis)					
Yi (30)	Track 1; retrospective cross-sectional	Ischemic stroke, recovery stage (*N* = 170)	Wind-phlegm-stasis; Qi-deficiency with collateral stasis; Liver-kidney deficiency	Manual eye-area inspection plus smartphone tongue imaging; ML secondary	Chinese AIS Guidelines 2018; TCM Internal Medicine Textbook 2021
Zheng and Fu (31)	Track 1; prospective descriptive single cohort	Hemorrhagic stroke, acute stage (*N* = 88)	Six TCM syndrome categories	Manual visual tongue examination by senior TCM physicians	CVD Guidelines 2005; National TCM Stroke Standards 1986
Gan et al. (32)	Track 1; retrospective cross-sectional	Ischemic stroke, recovery stage (*N* = 420)	Wind-phlegm; Qi-deficiency/blood stasis; Yin-deficiency; Phlegm-heat	Manual four-diagnosis with six tongue/pulse indices	TCM disease guideline 2008; SATCM clinical pathway 2017
Liu et al. (33)	Track 1; prospective serial assessment	Mixed ischemic and hemorrhagic stroke, acute stage (*N* = 328)	Tongue body/coating changes across six syndrome groups	Manual visual tongue diagnosis with serial observation	CT subtype plus national TCM guidelines
Xu et al. (34)	Track 1; syndrome-vs-syndrome case–control	Acute ischemic stroke (*N* = 1,035)	Qi-deficiency with blood stasis vs. other stroke syndromes	Multi-domain TCM clinical assessment including tongue/pulse/symptoms	Chinese AIS Guidelines 2023; ZY/T001-2022
Cao (35)	Track 1; prospective cross-sectional	Acute ischemic stroke (*N* = 150)	Five TCM syndrome categories	Manual tongue examination by three senior TCM physicians with standardized photography	Chinese AIS Guidelines 2018; National TCM Stroke Diagnostic Standard
Track 2: AI-assisted tongue diagnosis (narrative synthesis)					
Cui et al. (36)	Track 2; quantitative tongue-image analysis	Mixed ischemic and hemorrhagic stroke (*N* = 378)	Five TCM syndrome categories	Computerized RGB tongue-color extraction; no binary Se/Sp	ZY/T001.1–001 (1994/1996)
Wang et al. (37)	Track 2; AI model development	Mixed ischemic and hemorrhagic stroke (*N* = 284)	Eight TCM syndromes	DenseNet201 feature extraction + feature selection + Cubic SVM	CACM Stroke Disease Standard ZYYXH/T 22–2008
Wang (38)	Track 2; healthy-control case–control	Acute ischemic stroke vs. healthy controls (*N* = 300)	Qi-deficiency with blood stasis	ResNet18 and VGGNet16; VGGNet16 best	Chinese AIS Guidelines 2018; CACM Guidelines 2008
Han (39)	Track 2; healthy-control case–control	Acute ischemic stroke vs. healthy controls (*N* = 284)	Liver-Qi stagnation	ResNet18 and VGG16; ResNet18 best	GB/T16751.2–2021; Chinese AIS Guidelines 2018; CACM Guidelines 2008

AI, artificial intelligence; AIS, acute ischemic stroke; CVD, cerebrovascular disease; ICH, intracerebral hemorrhage; ML, machine learning; TCM, traditional Chinese medicine. Complete study-level extraction fields and footnotes are provided in Supplementary Table S5.

Detailed extraction fields, including age/sex distribution, complete syndrome categories, index-test details, AI-model notes, and reference-standard notes, are provided in Supplementary Table S5.

### 3.2 Risk of bias

Methodological quality varied between the two tracks (Figure 2). In Track 1, the reference standard domain raised the greatest concern: three studies were rated high risk, two unclear, and one low risk. The central concern was that composite TCM syndrome criteria could include, or did not clearly demonstrate independence from, tongue manifestations evaluated as index features, creating potential incorporation bias. In the index test domain, five studies were rated unclear risk and one high risk, mainly because blinding of the tongue assessor was not reported. Patient selection was rated low risk in two studies, unclear in three, and high in one. Flow and timing were rated low risk in all six studies. Applicability concerns were low across all three domains.

**Figure 2:**
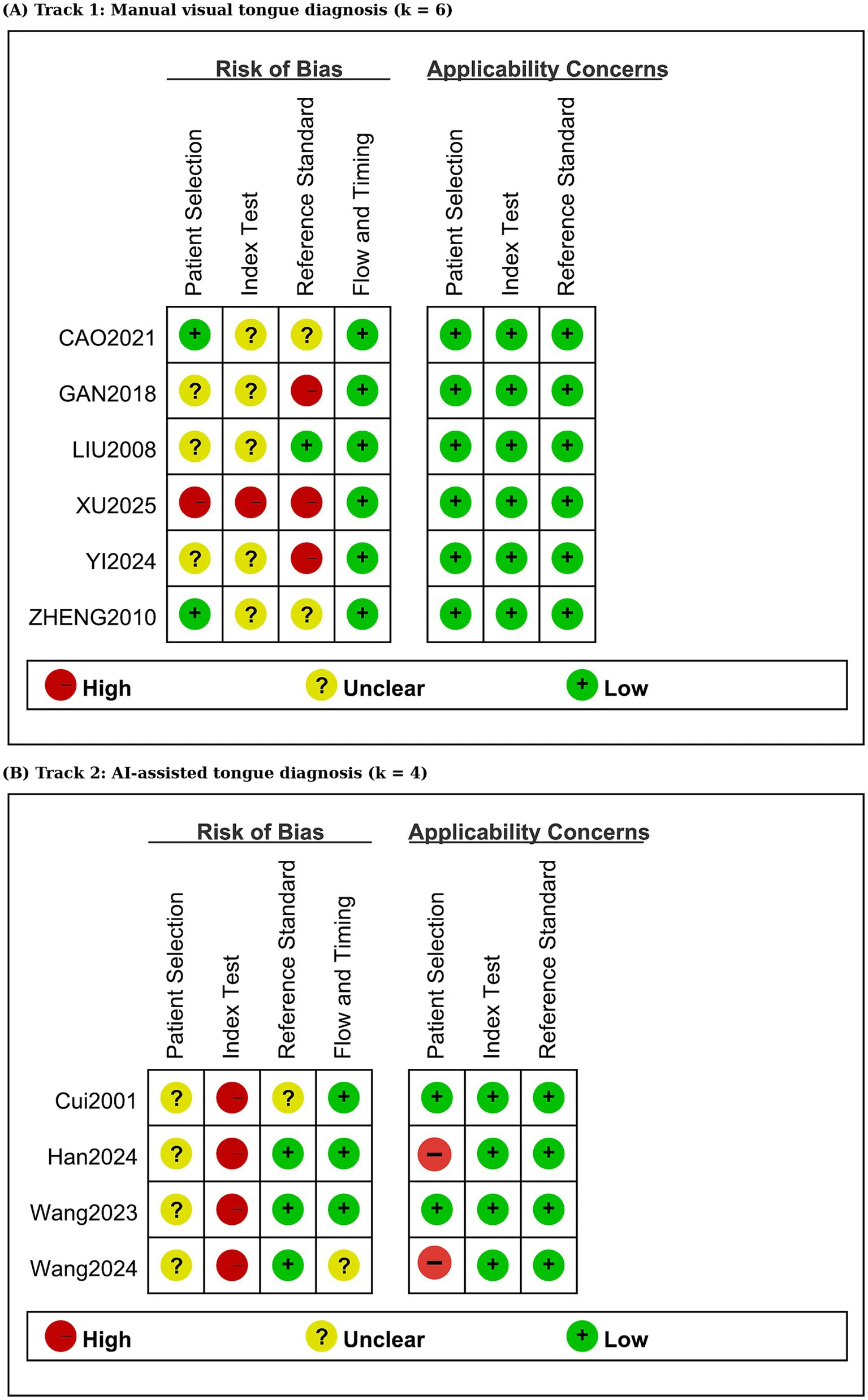
Risk of bias and applicability concerns assessed using QUADAS-2. **(A)** Track 1 (manual visual tongue diagnosis; *k* = 6). **(B)** Track 2 (artificial intelligence-assisted tongue diagnosis; *k* = 4). Risk of bias is presented across four domains (patient selection, index test, reference standard, flow and timing), along with applicability concerns for the first three domains.

In the four Track 2 studies, the index test domain was rated high risk in all cases, mainly because of post-hoc threshold optimization, image-level splitting or other possible data leakage, and the absence of external validation. Patient selection was rated unclear in all four studies. The reference standard was rated low risk in three and unclear in one. Flow and timing were rated low risk in three and unclear in one. The two healthy-control case–control AI studies were additionally interpreted as having serious spectrum and applicability limitations because they did not represent syndrome differentiation among patients with stroke in routine practice.

### 3.3 Exploratory diagnostic accuracy estimates of manual tongue diagnosis

Complete 2 × 2 contingency tables and heterogeneity statistics (*I*^2^, τ^2^) for each study and cluster were provided in Supplementary Table S6. The provenance, back-calculation, and rounding-residual audit for reconstructed or derived 2 × 2 rows was provided in Supplementary Table S9, and model-diagnostic, transformation-sensitivity, and affected-row exclusion analyses were provided in Supplementary Table S10. Figures 3, 4 presented paired univariate random-effects forest plots of pooled sensitivity and specificity for the six quantitatively synthesized clusters, displayed in two groups for readability, and Figure 5 presented the Greasy Coating bivariate SROC model. The forest plots displayed univariate random-effects estimates, whereas the Greasy Coating SROC summary point was derived from a bivariate random-effects model; these summaries were therefore not expected to be numerically identical. Results were presented below according to the four pre-defined TCM pathomechanical categories. Because independence between the index tongue feature and the composite reference standard was not assured across Track 1 studies, the pooled estimates were interpreted as provisional summary measures potentially affected by incorporation bias rather than definitive indicators of diagnostic performance. Accordingly, the following estimates were reported as exploratory and hypothesis-generating summaries, not as clinically validated diagnostic performance.

**Figure 3:**
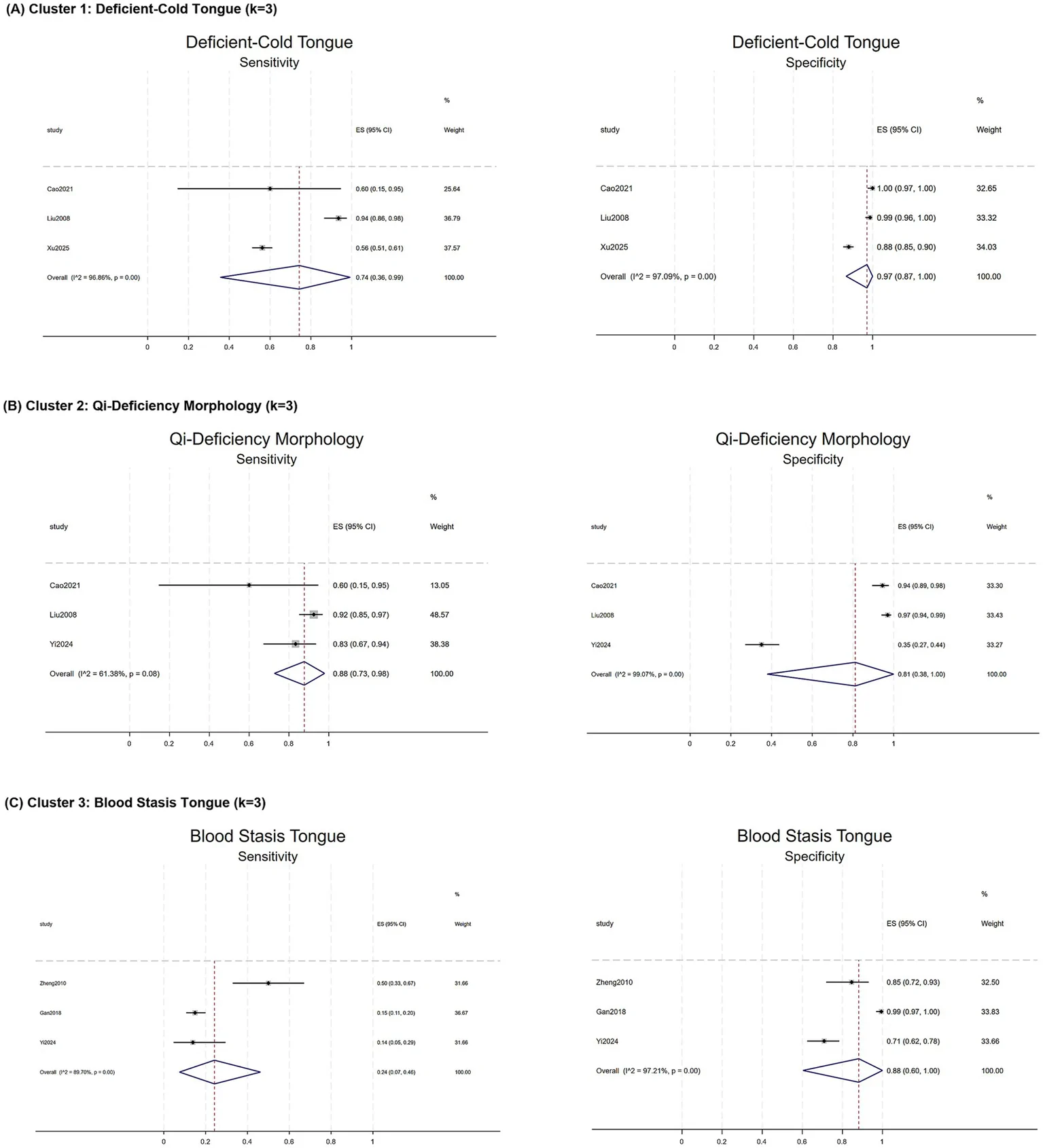
Univariate random-effects paired forest plots of sensitivity and specificity for Clusters 1–3: **(A)** Deficient-Cold Tongue; **(B)** Qi-Deficiency Morphology; and **(C)** Blood Stasis Tongue. Forest plots display descriptive univariate estimates using the Freeman–Tukey transformation with exact binomial study-level confidence intervals. For zero-cell studies, a 0.5 continuity correction was applied only in LR/DOR calculations, not to the univariate Se/Sp forest plots. Because reference-standard independence was not assured, these pooled estimates should be interpreted as provisional summaries of agreement with composite syndrome-classification processes rather than diagnostic accuracy against an independent syndrome truth.

**Figure 4:**
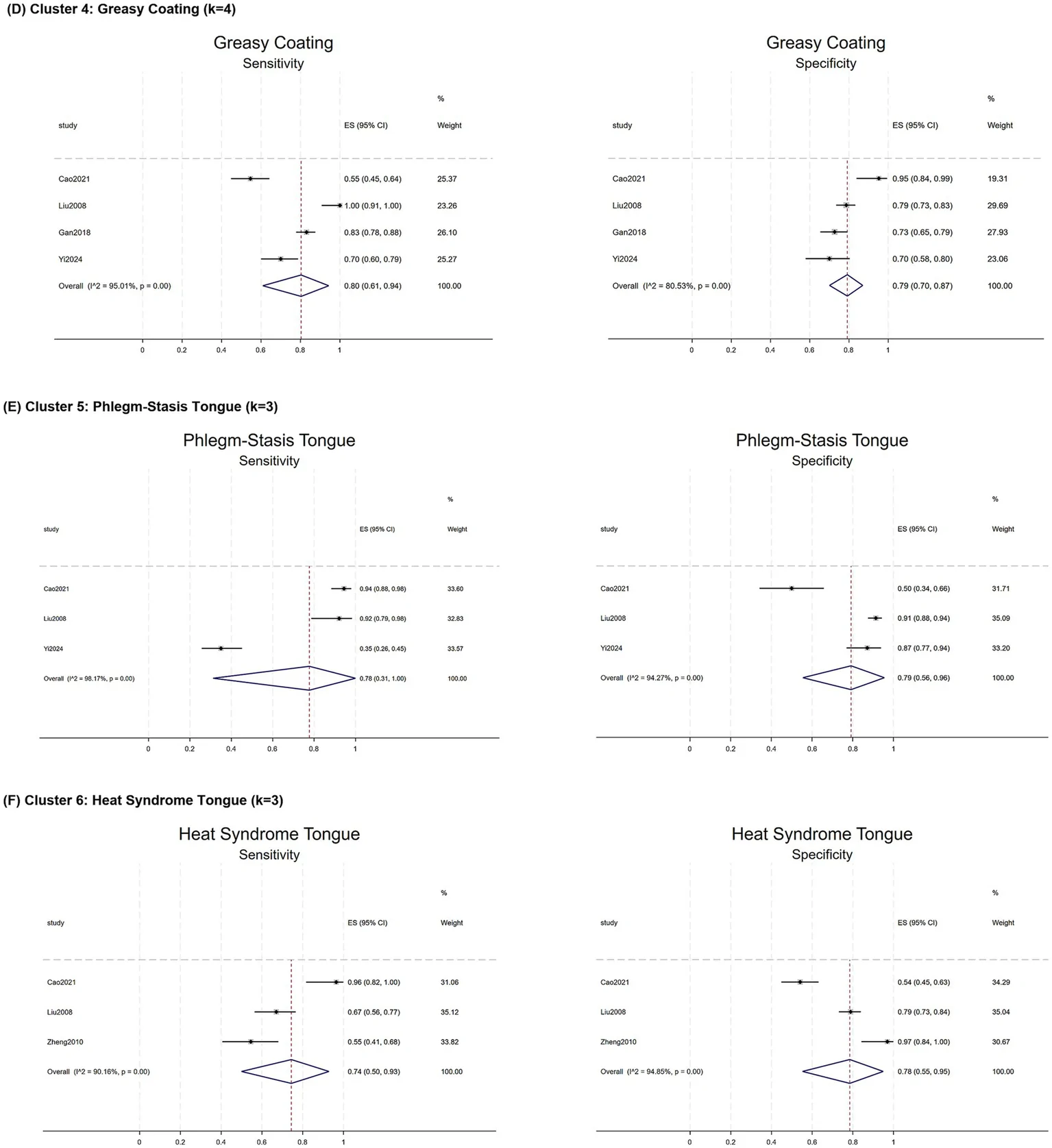
Univariate random-effects paired forest plots of sensitivity and specificity for Clusters 4–6: **(D)** Greasy Coating; **(E)** Phlegm-Stasis Tongue; and **(F)** Heat Syndrome Tongue. Panel lettering continues from Figure 3 to preserve the original six-cluster sequence. Forest plots display descriptive univariate estimates using the Freeman–Tukey transformation with exact binomial study-level confidence intervals. The Greasy Coating forest-plot summary was derived from univariate models and was therefore not expected to be numerically identical to the bivariate SROC summary operating point shown in Figure 5. Because reference-standard independence was not assured, these pooled estimates should be interpreted as provisional summaries of agreement with composite syndrome-classification processes rather than diagnostic accuracy against an independent syndrome truth.

**Figure 5:**
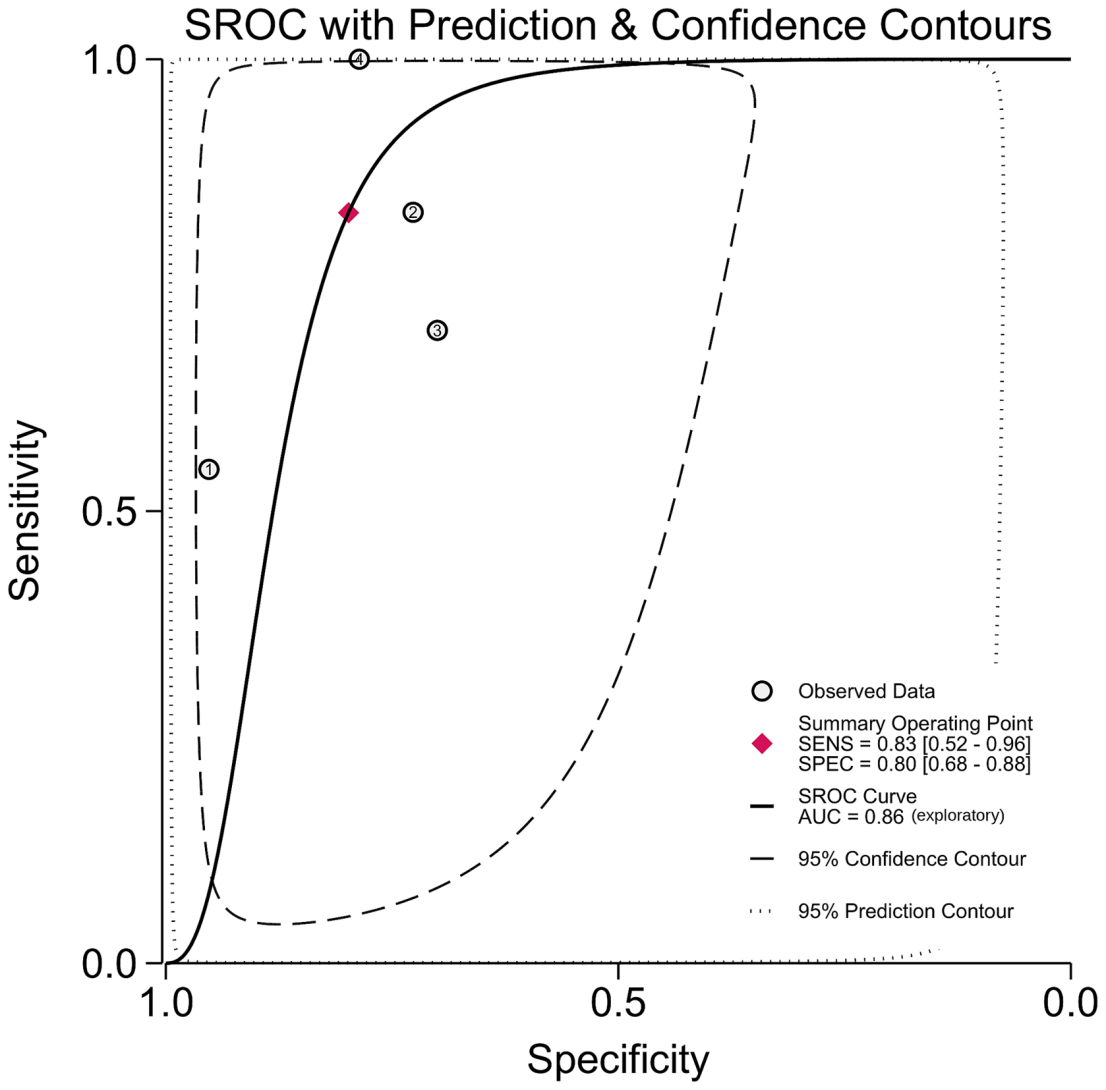
Bivariate SROC model for the Greasy Coating cluster (*k* = 4). The summary operating point was derived from a bivariate random-effects model, and the SROC x-axis is displayed with specificity decreasing from left to right. The AUC of 0.86 is presented as an exploratory point estimate. Because reference-standard independence was not assured, these model estimates should be interpreted as provisional summaries of agreement with composite syndrome-classification processes rather than diagnostic accuracy against an independent syndrome truth.

Wind-phlegm and Dampness syndrome. The Greasy Coating cluster (*k* = 4; *N* = 1,068) was the only cluster eligible for bivariate random-effects modeling. The bivariate model converged and yielded a summary operating point on the SROC curve with pooled sensitivity of 0.83 (95% CI: 0.52–0.96) and specificity of 0.80 (95% CI: 0.68–0.88), with a model-derived exploratory AUC point estimate of 0.86. The positive likelihood ratio was 4.1 (95% CI: 2.5–6.8), and the negative likelihood ratio was 0.21 (95% CI: 0.06–0.71). Because the model included only four studies, model diagnostics and GLMM sensitivity checks were reported in Supplementary Table S10 and the estimate was interpreted as exploratory. The corresponding univariate forest-plot summaries were sensitivity 0.80 (95% CI: 0.61–0.94; *I*^2^ = 95.01%) and specificity 0.79 (95% CI: 0.70–0.87; *I*^2^ = 80.53%). The Phlegm-Stasis Tongue cluster (*k* = 3; *N* = 648) showed sensitivity of 0.78 (95% CI: 0.31–1.00; *I*^2^ = 98.17%) and specificity of 0.79 (95% CI: 0.56–0.96; *I*^2^ = 94.27%), with a positive likelihood ratio of 3.8 (95% CI: 1.2–12.7) and a negative likelihood ratio of 0.20 (95% CI: 0.04–1.02).

Qi-deficiency and Blood-stasis syndrome. The Deficient-Cold Tongue cluster (*k* = 3; *N* = 1,513) had the numerically highest specificity point estimate among the pooled clusters. Sensitivity was 0.74 (95% CI: 0.36–0.99; *I*^2^ = 96.86%), and specificity was 0.97 (95% CI: 0.87–1.00; *I*^2^ = 97.09%). Because one study contained a zero false-positive cell, LR/DOR calculations used a 0.5 continuity correction only for that study. The positive likelihood ratio was 31.0 (95% CI: 2.8–341.6), and the negative likelihood ratio was 0.24 (95% CI: 0.07–0.88). The Qi-Deficiency Morphology cluster (*k* = 3; *N* = 648) yielded sensitivity of 0.88 (95% CI: 0.73–0.98; *I*^2^ = 61.38%) and specificity of 0.81 (95% CI: 0.38–1.00; *I*^2^ = 99.07%), with a positive likelihood ratio of 7.4 (95% CI: 0.76–71.7) and a negative likelihood ratio of 0.24 (95% CI: 0.07–0.85). The Blood Stasis Tongue cluster (*k* = 3; *N* = 678) showed sensitivity of 0.24 (95% CI: 0.07–0.46; *I*^2^ = 89.70%) and specificity of 0.88 (95% CI: 0.60–1.00; *I*^2^ = 97.21%), with a positive likelihood ratio of 2.83 (95% CI: 0.45–17.68), a negative likelihood ratio of 0.88 (95% CI: 0.65–1.19), and a DOR of 3.58 (95% CI: 0.35–36.50). These estimates indicated weak and unstable discriminatory performance rather than a reliable inverse diagnostic association. The Deficient-Cold PLR interval spanned more than two orders of magnitude, whereas the Qi-Deficiency Morphology PLR interval included unity; together with the extremely wide specificity interval for the latter, these estimates were incompatible with stable rule-in conclusions for either cluster.

Phlegm-heat and Excess Fire syndrome. The Heat Syndrome Tongue cluster (*k* = 3; *N* = 566) showed sensitivity of 0.74 (95% CI: 0.50–0.93; *I*^2^ = 90.16%) and specificity of 0.78 (95% CI: 0.55–0.95; *I*^2^ = 94.85%). The positive likelihood ratio was 2.86 (95% CI: 1.76–4.66), and the negative likelihood ratio was 0.42 (95% CI: 0.29–0.59).

Yin-deficiency syndrome. For the two Yin-Deficiency clusters (*k* = 2 each; *N* = 478), quantitative pooling was not performed because the number of studies was insufficient for stable variance estimation. In the Yin-Deficiency Coating cluster, raw individual-study sensitivity ranged from 0.67 to 1.00 (displayed as 0.67 to 0.99 after transformation in Figure 6) and specificity from 0.92 to 0.99. In the Yin-Deficiency Body cluster, sensitivity ranged from 0.60 to 0.78, and both studies reported specificity of 1.00. These data were presented as unpooled evidence maps in Figure 6.

**Figure 6:**
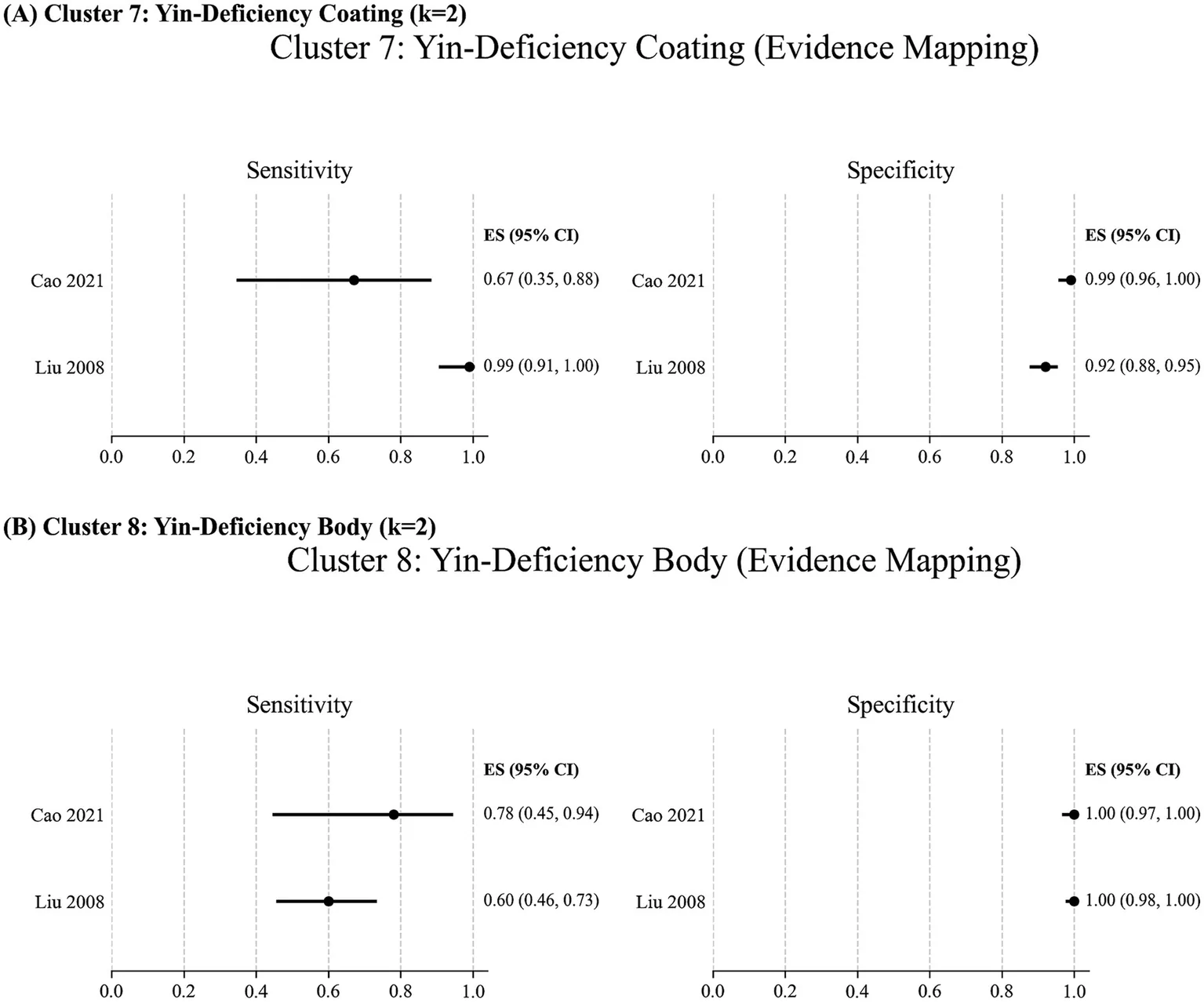
Evidence mapping of Yin-deficiency tongue feature clusters (*k* = 2 each): **(A)** Cluster 7, Yin-Deficiency Coating; **(B)** Cluster 8, Yin-Deficiency Body. Individual study estimates are presented without pooling due to the limited number of studies.

### 3.4 Narrative synthesis of artificial intelligence-assisted models

Four studies evaluated artificial intelligence-assisted tongue diagnosis for stroke syndrome differentiation (Table 3). The four studies used heterogeneous approaches, representing different stages of tongue-image analysis. Cui et al. (36) used computerized RGB color extraction within a tongue-diagnosis expert-system framework and reported significant color differences across five syndromes (*p* < 0.05), but did not pre-specify diagnostic thresholds or report individual-level classification performance. Wang et al. (37) used DenseNet201 feature extraction with feature selection and a Cubic SVM classifier for eight-class syndrome differentiation; based on 284 participants and 852 augmented images, the Cubic SVM classifier achieved 97.21% accuracy and 97.56% F1 score, with a minimum syndrome-level accuracy of 95.74%. Wang et al. (38) compared ResNet18 and VGGNet16 for Qi-deficiency and Blood-stasis identification in acute ischemic stroke versus healthy controls; VGGNet16 performed best, with accuracy of 90.67%, recall of 85.60%, specificity of 94.29%, and F1 score of 88.43%. Han et al. (39) compared ResNet18 and VGG16 for Liver-Qi stagnation identification in acute cerebral infarction versus healthy controls; ResNet18 performed best, with accuracy of 91.25%, recall of 86.76%, specificity of 94.13%, and F1 score of 88.60%.

**Table 3:** STARD-AI/TRIPOD+AI-informed concise audit of AI-assisted tongue diagnosis studies (*k* = 4), including two stroke-syndrome classification studies and two healthy-control case–control studies retained only for narrative and applicability appraisal.

Study	AI approach and diagnostic task	Dataset and acquisition reporting	Image-to-participant ratio and split level	Internal performance and validation status	Comparator, calibration, and external-validation audit	Applicability, leakage, and future-validation interpretation
Cui et al. (36)	RGB-based expert-system/quantitative color-feature analysis; five-syndrome differentiation among patients with stroke.	378 stroke patients; digital tongue images acquired using an OLYMPUS digital camera and processed in a TCM tongue-diagnosis expert system; additional lighting/white-balance details NR.	Image count not separately reported; image-to-participant ratio not estimable; no train/test split or patient-level prediction validation reported.	Internal group-level quantitative analysis; significant RGB-derived group differences; no individual-level binary or multiclass accuracy metrics reported.	No simple baseline model or head-to-head human expert comparator reported; calibration, decision-curve analysis, and independent external validation NR.	Early quantitative color-analysis proof-of-concept; no prespecified diagnostic threshold and limited acquisition reporting; independence of syndrome labels from tongue features not demonstrated.
Wang et al. (37)	DenseNet201 feature extraction + feature selection + Cubic SVM; eight-class stroke syndrome classification among patients with stroke.	284 stroke patients; acquisition hardware, lighting, and white-balance protocol NR; images increased to 852 after augmentation.	852 augmented images/284 participants (approximately 3 images per participant after augmentation); internal 3-fold and 6-fold cross-validation; patient-level separation for augmented images NR.	Cubic SVM accuracy 97.21%, F1 score 97.56%; lowest syndrome-level accuracy 95.74%; no independent external or multicenter validation reported.	No simple baseline model or head-to-head human expert comparator reported; calibration and decision-curve analysis NR; external validation NR.	Promising internal multiclass performance, but interpretation is limited by augmentation, incomplete acquisition reporting, and possible optimism if augmented images or feature selection were not confined within patient-level folds.
Wang (38)	ResNet18 and VGGNet16 CNNs; Qi-deficiency and Blood-stasis in acute ischemic stroke vs. healthy controls.	300 participants; 3,000 images; relatively detailed digital-camera protocol reported, including camera/lens, gray-card/manual white balance, controlled lighting, posture, and quality control.	3,000 images/300 participants = 10 images per participant; internal image-level 80/20 split (2,400 training, 600 testing); patient-level split NR.	VGGNet16 best: accuracy 90.67%, recall 85.60%, specificity 94.29%, F1 score 88.43%; no independent external validation reported.	No simple baseline model or head-to-head human expert comparator reported; calibration and decision-curve analysis NR; external validation NR.	Healthy-control comparator does not directly evaluate syndrome differentiation among patients with stroke; multiple images per participant with image-level splitting raised possible leakage/pseudo-replication concerns; retained only as Track 2 narrative/applicability evidence.
Han (39)	ResNet18 and VGG16 CNNs; Liver-Qi stagnation in acute cerebral infarction vs. healthy controls.	284 participants; 2,840 images; relatively detailed digital-camera acquisition protocol reported; exact camera/lens model NR.	2,840 images/284 participants = 10 images per participant; internal image-level 80/20 split (2,280 training, 560 testing); patient-level split NR.	ResNet18 best: accuracy 91.25%, recall 86.76%, specificity 94.13%, F1 score 88.60%; no independent external validation reported.	No simple baseline model or head-to-head human expert comparator reported; calibration and decision-curve analysis NR; external validation NR.	Healthy-control comparator does not directly evaluate syndrome differentiation among patients with stroke; multiple images per participant with image-level splitting raised possible leakage/pseudo-replication concerns; retained only as Track 2 narrative/applicability evidence.

The table summarizes AI approach and diagnostic task, dataset and acquisition reporting, image-to-participant ratio and split level, reported internal performance, comparator/calibration/external-validation status, and applicability/leakage interpretation. Full model-level methodology, image-acquisition details, preprocessing, augmentation, and detailed reporting/applicability appraisal are provided in Supplementary Table S7.

Taken together, these studies provided early proof-of-concept evidence that tongue images could be processed into quantitative or learned image features for syndrome-related classification; however, their clinical applicability remained limited by validation design, comparator selection, and reporting quality. None reported independent external validation or multicenter validation, calibration assessment, decision-curve analysis, or a head-to-head comparison with independent expert TCM physicians under the same clinical task. The two 2024 deep-learning case–control studies compared acute ischemic stroke syndrome cases with healthy volunteers, creating serious spectrum bias (40) and indirectness for the intended clinical task of syndrome differentiation among patients with stroke. Image-acquisition reporting also varied across studies: the two 2024 studies reported relatively detailed digital-camera acquisition protocols, including white-balance control, illumination control, posture instructions, and exclusion of poor-quality images, whereas the earlier RGB-based and DenseNet/SVM studies reported fewer acquisition details. The image-to-participant ratios and validation structures also limited interpretation: Wang et al. reported 852 augmented images from 284 participants, whereas Wang ZY et al. and Han et al. reported 3,000 images from 300 participants and 2,840 images from 284 participants, respectively. Because patient-level splitting was not clearly reported for these image-rich datasets, image-level leakage or pseudo-replication could not be excluded. In the two 2024 deep-learning studies, image-level train/test splitting and the use of multiple images per participant also raised possible data-leakage concerns. No AI-assisted study reported calibration assessment or decision-curve analysis for model evaluation (41). Table 3 provided a concise STARD-AI/TRIPOD+AI-informed audit of model architecture, diagnostic task, dataset and acquisition reporting, image-to-participant ratio, split level, internal performance, comparator/calibration/external-validation status, and applicability/leakage interpretation; complete model-level methodology and detailed reporting/applicability appraisal were provided in Supplementary Table S7.

### 3.5 Sensitivity analysis and illustrative post-test probability analysis

Leave-one-out sensitivity analyses showed that several clusters were sensitive to single-study effects and were interpreted as exploratory. In the Blood Stasis Tongue cluster, exclusion of Yi 2024 increased specificity to 0.98 (95% CI: 0.95–0.99) while sensitivity remained low, indicating that the main finding was unstable and influenced by study composition. In the Qi-Deficiency Morphology and Greasy Coating clusters, specificity estimates varied materially across leave-one-out iterations. Complete results were provided in Supplementary Table S4. Additional methodological sensitivity analyses supported cautious interpretation rather than strong robustness claims. Logit-transformed analyses produced broadly similar point estimates to the Freeman-Tukey analyses, although confidence intervals remained wide under high heterogeneity. After Gan 2018 was excluded, only three studies remained; therefore, the Greasy Coating bivariate model was not refitted, and corresponding univariate Freeman-Tukey and logit checks were reported instead. The 2 × 2 provenance and rounding-residual audit was provided in Supplementary Table S9, and the methodological sensitivity analyses were provided in Supplementary Table S10.

Fagan nomograms (25) for two illustrative clusters were shown in Figure 7. Under a hypothetical pre-test probability of 50%, the Deficient-Cold Tongue cluster (PLR = 31.0) would increase the modeled post-test probability to approximately 97%, whereas the negative pathway (NLR = 0.24) would reduce it to approximately 19%. Under the same hypothetical scenario, the Greasy Coating cluster (bivariate LR + = 4.1; LR = 0.21) would increase the modeled post-test probability to approximately 80%, whereas the negative pathway would reduce it to approximately 18%. These post-test probability estimates were interpreted as illustrative scenario analyses rather than evidence of clinical utility, because the 50% pre-test probability was hypothetical, syndrome prevalence varied across studies, and the certainty of evidence was very low. Three-scenario analyses at pre-test probabilities of 20, 50, and 80% were provided in Supplementary Table S8.

**Figure 7:**
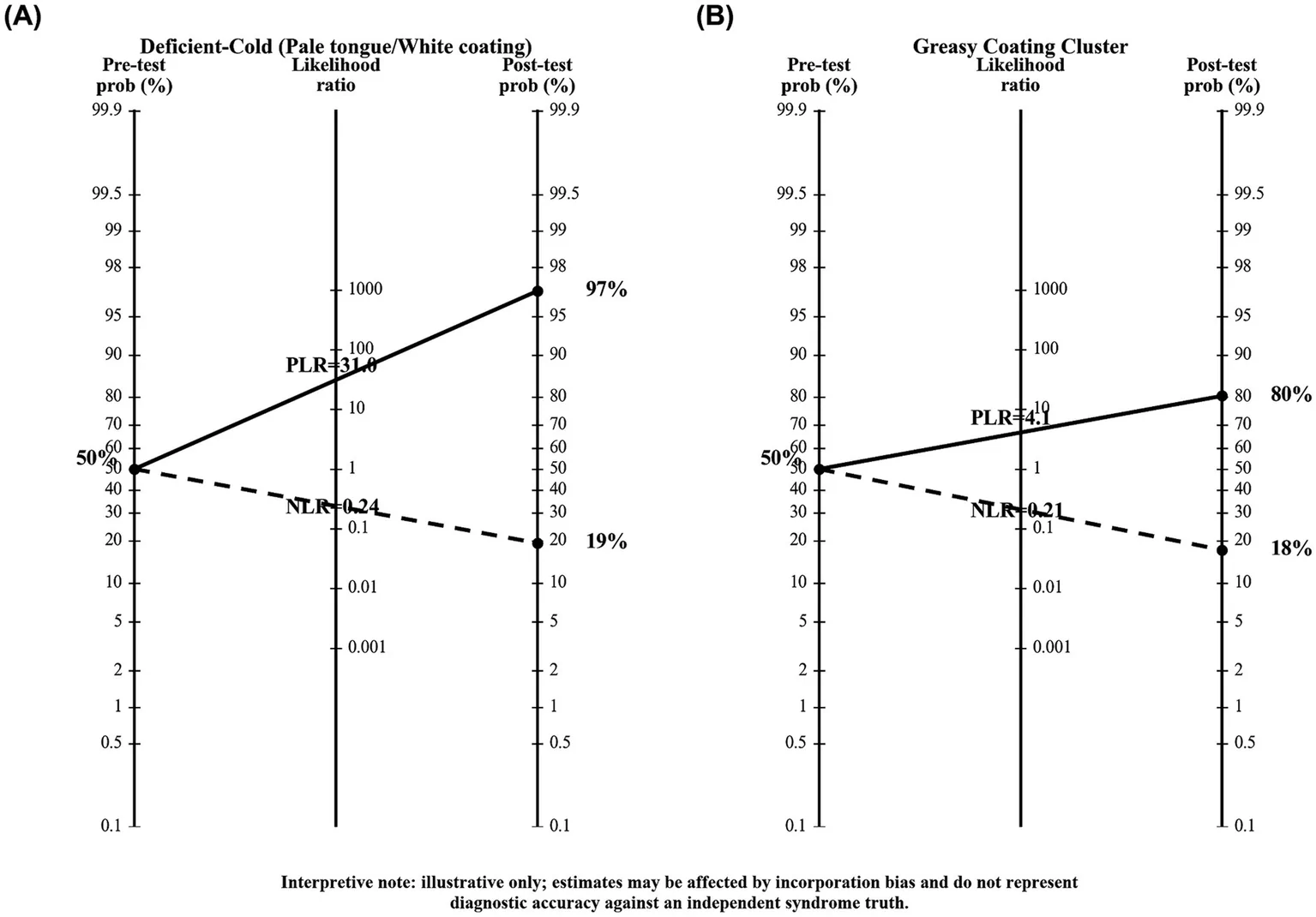
Fagan nomograms illustrating post-test probability changes. Panel **(A)**: Deficient-Cold Tongue cluster. Panel **(B)**: Greasy Coating cluster. Nomograms depict post-test probabilities derived from point-estimate likelihood ratios at a pre-test probability of 50%; values are illustrative because syndrome prevalence in real clinical settings remains uncertain. They do not establish clinical utility because the certainty of evidence was very low, reference-standard independence was not assured, and the modeled probabilities should not be interpreted as diagnostic accuracy against an independent syndrome truth or as clinical decision thresholds.

### 3.6 Certainty of evidence and reporting bias

Deeks’ funnel plot asymmetry test (26) was not performed because all diagnostic clusters contained fewer than ten studies, rendering the test statistically underpowered. A potential language bias favoring Chinese-language literature could not be excluded, although three English-language and three Chinese-language databases were searched. The certainty of evidence was rated Very Low across all eight tongue feature clusters (Table 1). Risk of bias accounted for downgrading by two levels, primarily because independence of the evaluated tongue feature from the composite reference standard was not assured, raising serious concern for incorporation bias (7). Indirectness warranted downgrading by one level because all included studies were conducted in mainland China and the evidence base was geographically, culturally, clinically, and healthcare-system specific. Inconsistency warranted downgrading by one to two levels, reflecting substantial between-study heterogeneity that may have arisen from differences in visual thresholds, syndrome definitions, stroke subtype or stage, and study design. Imprecision warranted downgrading by one to two levels because of wide confidence intervals and sparse evidence networks. For the two Yin-Deficiency clusters, inconsistency could not be formally assessed because only two studies were available.

## 4 Discussion

### 4.1 Diagnostic asymmetry and the morphology–color gradient

Across the six quantitatively synthesized tongue-feature clusters, summary sensitivity estimates ranged from 0.24 to 0.88 and specificity estimates from 0.78 to 0.97; the certainty of evidence was Very Low across all eight clusters. The cross-cluster pattern appeared asymmetrical: selected tongue features showed exploratory evidence of greater rule-in than rule-out potential, but these findings should not be interpreted as clinically validated diagnostic adjuncts (42). Because reference-standard independence was not assured across the evidence base, the estimates are more appropriately interpreted as provisional agreement with current syndrome-classification processes than as diagnostic accuracy against an independent syndrome truth. This systematic review is, to our knowledge, the first to apply formal GRADE-DTA methodology (18) to evaluate tongue manifestations for TCM syndrome differentiation in patients with stroke. The sharp attrition from 13,895 identified records to 10 eligible studies is itself informative. It indicates that the post-stroke tongue-diagnosis literature is large in volume but thin in diagnostically testable evidence: most publications could not support 2 × 2 table reconstruction, likelihood-ratio estimation, or certainty appraisal within a diagnostic accuracy framework. Thus, the sparse analyzable evidence base should not be interpreted only as a limitation of this review, but also as a finding about the current methodological maturity of the field.

Within the constraints of very low-certainty evidence, the Deficient-Cold Tongue cluster had a specificity point estimate of 0.97 and a PLR point estimate of 31.0. However, the PLR confidence interval ranged from 2.8 to 341.6, spanning more than two orders of magnitude, while sensitivity and specificity were highly heterogeneous across only three studies. Although the PLR interval did not include unity, its width was incompatible with a stable estimate of the magnitude of discrimination and did not justify ranking this cluster as a reliable rule-in feature. This numerically elevated but highly imprecise estimate should be viewed as an exploratory hypothesis, and it may also partly reflect the diagnostic weight assigned to pale or swollen tongue morphology within the composite TCM reference standards. Sensitivity was only 0.74, meaning approximately one in four true cases would be missed if tongue morphology alone were used as the entry criterion. Thus, this finding is best interpreted as a hypothesis-generating observation requiring future validation within the broader four-diagnosis framework, not as evidence that tongue morphology can independently define the syndrome.

A further, more tentative finding was a possible morphology-color gradient: in this sparse evidence base, morphology and coating-related features had numerically higher rule-in point estimates than color-dominant features. This should not be interpreted as a stable biological hierarchy. It may reflect measurement properties: body size, teeth-mark indentation, and coating thickness may be easier for practitioners to categorize consistently than purple hue, crimson redness, or yellow coating intensity, which are more sensitive to lighting, camera settings, and individual color perception. A further possibility is that morphology-related findings may carry greater implicit weight within composite syndrome criteria, thereby amplifying incorporation effects when those same features are evaluated as index tests. Because formal feature weights were not reported, this explanation remains hypothetical. The observed gradient is therefore a hypothesis about measurement reliability and classification structure, not a validated ordering or stable ranking of pathophysiological or diagnostic importance.

### 4.2 Exploratory diagnostic value across specific syndrome clusters

The following paragraphs summarize exploratory diagnostic findings at the individual syndrome level. For Qi-deficiency and blood-stasis syndrome, two clusters provided complementary but uncertain information. Neither cluster supported a stable diagnostic conclusion. The Deficient-Cold Tongue cluster had numerically high specificity and PLR point estimates, but the PLR confidence interval was extremely wide. The Qi-Deficiency Morphology cluster had a relatively high sensitivity point estimate, but its specificity was highly uncertain and its PLR confidence interval included unity. Taken together, a pale, enlarged tongue body with teeth marks may be associated with current composite syndrome classification, but the very low GRADE certainty and the shared incorporation-bias mechanism mean these estimates require validation against an index-feature-independent reference process before clinical application can be considered.

For wind-phlegm and dampness syndrome, the Greasy Coating cluster (*k* = 4) yielded bivariate sensitivity and specificity point estimates of 0.83 and 0.80, respectively. However, these estimates were based on only four studies and remained uncertain because of wide confidence intervals, substantial heterogeneity, sparse-model instability, and possible incorporation effects. The available evidence therefore did not establish a stable or clinically distinct diagnostic role for greasy coating. The Phlegm-Stasis Tongue cluster produced similarly moderate point estimates but with wider uncertainty, providing no secure evidence of a clinically distinct diagnostic role.

The Blood Stasis Tongue cluster showed weak and unstable discriminatory performance, with low sensitivity, high heterogeneity, and wide likelihood-ratio confidence intervals crossing the null. Its instability in leave-one-out analysis indicates that the apparent direction and magnitude of discrimination are strongly dependent on study composition. Differences in ischemic versus hemorrhagic stroke, acute versus recovery-stage assessment, and syndrome classification procedures are plausible contributors, but the available data are too sparse to separate these explanations. Future studies should stratify by stroke etiology and stage before evaluating tongue features for blood stasis.

For yin-deficiency syndrome, quantitative pooling was not feasible because only two studies were available. Although the individual studies reported high specificity (0.92–1.00), these values cannot be treated as a stable pooled property of the cluster, particularly in the presence of possible incorporation bias. Sensitivity was lower or variable (0.60–0.78 for the body cluster; approximately 0.67–1.00 for the coating cluster), indicating uncertain screening value. The paucity of data makes this the least well-characterized syndrome cluster in the current review, and further primary studies using independent adjudication are needed.

The Heat Syndrome Tongue cluster had a PLR of 2.86, indicating only modest incremental information under the current study designs. This result does not establish a specific diagnostic role for tongue color in practice; rather, it suggests that a color-dominant feature alone is unlikely to provide strong rule-in evidence for phlegm-heat syndrome. Its added value should be tested prospectively against independent syndrome adjudication and in combination with other pre-specified clinical findings.

### 4.3 Incorporation bias and the limits of current evidence

Despite the numerically notable likelihood ratios observed for some clusters, the certainty of evidence was rated Very Low across all clusters. The primary methodological threat was potential incorporation bias in the reference standard domain (7). In the available study descriptions, the composite TCM syndrome standards included, or did not clearly demonstrate independence from, the tongue manifestations evaluated as index features. This creates a structural interpretive problem: where an index feature contributes to syndrome classification, the index test and reference standard are non-independent. Under such circumstances, sensitivity and specificity may be inflated to an unknown degree, and no meta-analytic adjustment can restore the missing independence. This interpretive constraint applies to the forest plots (Figures 3, 4), the Greasy Coating SROC model (Figure 5), the GRADE-DTA absolute effects (Table 1), and the Fagan nomograms (Figure 7), which are intended to make the available evidence transparent rather than to establish clinically validated diagnostic thresholds.

This problem is not unique to our review; it applies whenever a single TCM diagnostic sign is evaluated against a composite syndrome standard that includes, or may include, that same sign. Resolving it will require a genuine methodological shift. One approach is to use a reference-standard process independent of the index tongue feature: neuroimaging should confirm stroke subtype and relevant clinical characteristics, while a blinded TCM expert panel adjudicates syndrome status using pre-specified criteria that exclude the feature under evaluation. A second approach is Bayesian latent class analysis, which can estimate diagnostic parameters without assuming a perfect reference standard. No feature-independent adjudication procedure was identified in the extracted study information. Accordingly, the pooled estimates in this review should be interpreted as descriptive, hypothesis-generating summaries requiring prospective evaluation against an index-feature-independent reference process, not as evidence of independently validated biomarkers.

### 4.4 Manual versus AI-assisted approaches and future directions

The two diagnostic tracks examined in this review face complementary limitations. Manual visual assessment is clinically accessible and integrates naturally with the broader four-diagnosis framework, but it is vulnerable to inter-observer variation in visual thresholds. Such variation is one plausible contributor to the substantial heterogeneity observed across most clusters; other likely contributors include differences in stroke subtype and stage, patient spectrum, syndrome definitions, and study design. As with any study-level synthesis, these summaries cannot resolve patient-level heterogeneity or ecological inference problems (43). Leave-one-out analyses confirmed that removing a single study frequently shifted pooled sensitivity or specificity by clinically meaningful margins, underscoring that the pooled estimates should be treated as hypothesis-generating summaries rather than stable clinical thresholds.

Artificial intelligence-assisted models offer algorithmic consistency in threshold application, which in principle could reduce inter-observer variability. However, algorithmic consistency does not by itself solve the more fundamental reference-standard problem. If the training labels are derived from composite TCM syndrome criteria that already include tongue manifestations, an AI model may learn to reproduce the same incorporated standard more efficiently rather than discover an independent syndrome signal. In addition, the four Track 2 studies included in this review all suffered from serious methodological shortcomings: spectrum bias from artificially balanced datasets, the absence of independent external validation, and a complete lack of calibration or decision-curve analysis (41). None reported whether their models’ predicted probabilities corresponded to actual syndrome prevalence in real clinical populations. Current AI models also face hardware-level domain shift (44): models trained on images captured under standardized laboratory conditions may fail when deployed in actual clinical environments with variable smartphone sensors and ambient lighting.

The included AI studies should therefore be interpreted as heterogeneous early-stage investigations rather than as a single body of validated prediction models. RGB-based approaches quantify explicit color features, DenseNet/SVM pipelines combine deep feature extraction with conventional machine-learning classification, and ResNet/VGG-family convolutional networks learn higher-dimensional image representations directly from tongue photographs. Apparent differences in reported performance should not be interpreted as evidence that one architecture is clinically superior, because the studies differed in diagnostic task, sample composition, data augmentation, acquisition reporting, comparator design, and validation strategy.

Image acquisition is another likely determinant of model transportability. Tongue-image models may be sensitive to camera hardware, illumination, white balance, posture, background, and image-quality control. These factors may contribute to domain shift and should be reported in sufficient detail.

A further critical gap was the absence of human-standard comparator evaluation. No included AI study compared model performance against independent expert TCM physicians under the same clinical task. Therefore, high reported internal accuracy should be interpreted as reproduction of study-specific reference labels rather than evidence that AI improves clinical syndrome differentiation. Because these labels may themselves incorporate tongue manifestations, AI models may also inherit the circularity present in manual diagnostic studies. The two healthy-control case–control studies were therefore retained only as Track 2 narrative and applicability evidence, and were not treated as direct evidence for syndrome differentiation among patients with stroke.

Looking forward, the most urgent need is not for more sophisticated algorithms alone but for better study design. Future diagnostic accuracy studies--whether manual or AI-assisted--should employ independent syndrome adjudication, enroll prospective multicenter cohorts that reflect real-world syndrome prevalence, stratify by stroke subtype, and report calibration alongside discrimination. For AI models specifically, external validation on data from institutions and imaging devices not involved in model training is essential before any claims of clinical utility can be made. Such validation should test whether model performance remains stable across differences in image-acquisition protocols, camera hardware, ambient lighting, color calibration, syndrome-labeling practices, patient spectrum, syndrome prevalence, and healthcare-system context. Building on these external-validation requirements, future AI studies should also use patient-level data splits, pre-specified syndrome labels, calibration assessment, decision-curve analysis, human-comparator benchmarking, and clinically relevant stroke-syndrome comparators. Multi-modal approaches that integrate tongue imaging with structured clinical data such as NIHSS scores and neuroimaging findings may eventually be evaluated for whether they improve syndrome-classification support, but such systems must first demonstrate added value over standard clinical assessment in prospective head-to-head comparisons.

An additional and distinct evidence gap concerns patient-centered outcomes. Even if future studies establish index-feature-independent agreement between tongue-based classification and TCM syndrome adjudication, such agreement would not by itself demonstrate prognostic or clinical utility. The included evidence did not establish whether manual or AI-assisted tongue-based syndrome classifications were associated with mortality, 90-day modified Rankin Scale scores, stroke recurrence, early neurological deterioration, or functional recovery, nor whether using these classifications to guide treatment improved any of these outcomes. Accordingly, the present findings should not be used to infer benefit for clinical decision-making or patient outcomes. Future prospective cohorts should pre-specify patient-centered endpoints and assess incremental prognostic value beyond established clinical and neuroimaging predictors; where syndrome classification is used to guide management, prospective impact studies should test whether it improves outcomes compared with standard care.

### 4.5 Strengths and limitations

This review has several methodological strengths. Rather than providing definitive accuracy estimates, the main contribution of this review is to show, after a broad bilingual search, how little of the large tongue-diagnosis literature currently meets the minimum requirements for diagnostic-accuracy evaluation. It is the first to apply GRADE-DTA certainty assessment to tongue-based diagnostic accuracy data in patients with stroke. The dual-track design allowed separate synthesis of manual and artificial intelligence-assisted studies using appropriate methods for each. Pre-specified clinical clustering based on TCM pathomechanical categories avoided post-hoc data-driven grouping. The unit-of-analysis decision for overlapping cohorts (Cao 2021 thesis versus journal article) (35) was documented prospectively. Statistical model selection followed a hierarchical rule that prioritized the bivariate model when feasible and downgraded to univariate models only when sparse data made bivariate modeling unstable.

Several limitations should be acknowledged. First and most importantly, reference-standard independence could not be assured across Track 1 studies: the reported composite TCM syndrome criteria included, or did not clearly exclude, tongue manifestations evaluated as index features. This creates serious potential for incorporation bias; consequently, the pooled accuracy estimates should be viewed primarily as provisional agreement with composite syndrome classification processes rather than as diagnostic accuracy against an independent syndrome truth.

Second, all included studies were conducted in mainland China, which substantially limits cross-cultural, clinical, and healthcare-system generalizability. For manual tongue diagnosis, cultural and diagnostic-context differences may affect applicability because TCM syndrome differentiation in mainland China is routinely taught, guideline-informed, and embedded within the broader four-diagnosis framework; in other settings, practitioner training, operational definitions of syndromes, and the relative diagnostic weight assigned to tongue inspection may differ. Clinical differences may further limit transferability because patient case-mix, stroke subtype and stage, comorbidity profiles, medication exposure, oral hygiene, hydration or nutritional status, and post-stroke rehabilitation status may influence tongue body, coating, and color manifestations. At the healthcare-system level, the role of TCM in stroke care, the pre-test probability of specific TCM syndromes, and the extent to which tongue findings inform treatment decisions may differ between mainland Chinese TCM-integrated stroke pathways and non-mainland Chinese, non-Asian, or Western integrative-medicine settings.

For AI-assisted models, cultural and diagnostic-context differences may affect generalization through site-specific syndrome-labeling practices and local syndrome-classification standards. Clinical differences may affect model transportability through shifts in patient spectrum, syndrome prevalence, stroke subtype and stage, and comorbidity profiles. Healthcare-system differences may further affect real-world deployment because image-acquisition workflows, camera hardware, ambient lighting, color calibration, clinical documentation, and decision-making pathways may differ from the development setting. Therefore, the current manual tongue-diagnosis estimates and reported AI model performances should not be assumed to transfer directly to other populations or healthcare systems without prospective external validation.

Third, despite searching three English-language and three Chinese-language databases, a potential language bias favoring Chinese-language literature cannot be excluded. Fourth, formal assessment of publication bias using Deeks’ test (26) was not performed because all clusters contained fewer than ten studies. Fifth, quantitative pooling was not performed for the two Yin-Deficiency clusters (*k* = 2) to avoid unreliable variance estimation. Sixth, design-stratified sensitivity analysis was not feasible because the small number of studies within each cluster (*k* = 3–4) precluded meaningful subgroup modeling. The Greasy Coating bivariate model was fitted with only four studies, and several univariate estimates were close to the probability boundary. Although model diagnostics, GLMM comparison, logit-transformed sensitivity analyses, and affected-row exclusion checks were added, these analyses cannot remove the instability inherent in sparse evidence networks. Seventh, Track 2 AI studies used small, single-center datasets and, in two studies, healthy controls rather than stroke syndrome controls.

## 5 Conclusion

This GRADE-DTA review shows that very little of the large tongue-diagnosis literature meets the minimum requirements for formal diagnostic-accuracy evaluation. The manual evidence was of very low certainty and did not support stable cluster-specific diagnostic conclusions; because reference-standard independence was not assured, the estimates should be interpreted as provisional agreement with existing syndrome-classification processes rather than diagnostic accuracy against an independent syndrome truth. These findings do not establish manual tongue diagnosis as a clinically validated or standalone diagnostic tool, and the four AI-assisted studies remain early-stage because they lacked independent external validation and calibration assessment. Future studies should use prospective multicenter diagnostic-accuracy designs with independent syndrome adjudication, transparent image-acquisition protocols, stroke-subtype stratification, and external validation across devices, populations, and healthcare settings.

## Data Availability

The original contributions presented in the study are included in the article/Supplementary material, further inquiries can be directed to the corresponding author.
