# Supplementary material for: Diagnostic accuracy of tongue diagnosis for stroke syndrome differentiation in traditional Chinese medicine: a systematic review, meta-analysis, and narrative synthesis of artificial intelligence-assisted models
Source: Front Neurol. 2026 Oct 7;17:1874937. doi: 10.3389/fneur.2026.1874937 (PMC13683162; doi:10.3389/fneur.2026.1874937)
Supplement: Supplementary file 1 [file Data_Sheet_1.pdf]

Supplementary Material

Diagnostic accuracy of tongue diagnosis for stroke syndrome differentiation in traditional Chinese medicine: a systematic review, meta-analysis, and narrative synthesis of artificial intelligence-assisted models

Supplementary Appendix S1: Full search strategies for all databases

All databases were searched from inception to 23 February 2026. No language restrictions were applied.

PubMed (searched 23 February 2026)

("Stroke"[MeSH Terms] OR "Stroke"[Title/Abstract] OR "Cerebrovascular Accident"[Title/Abstract] OR "Cerebrovascular Accidents"[Title/Abstract] OR "CVA"[Title/Abstract] OR "CVAs"[Title/Abstract] OR "Cerebral Infarction"[Title/Abstract] OR "Cerebral Stroke"[Title/Abstract] OR "Apoplexy"[Title/Abstract] OR "Brain Ischemia"[Title/Abstract] OR "Cerebral Hemorrhage"[Title/Abstract] OR "Cerebrovascular Disorders"[Title/Abstract])

AND

("Tongue"[MeSH Terms] OR "medicine, chinese traditional"[MeSH Terms] OR "diagnosis, computer assisted"[MeSH Terms] OR "Tongue diagnosis"[Title/Abstract] OR "Glossoscopy"[Title/Abstract] OR "Tongue image"[Title/Abstract] OR "Tongue manifestation"[Title/Abstract] OR "Tongue appearance"[Title/Abstract] OR "Tongue coating"[Title/Abstract] OR "Tongue fur"[Title/Abstract] OR "Tongue body"[Title/Abstract] OR "Tongue proper"[Title/Abstract] OR "Digital tongue"[Title/Abstract] OR "Automated tongue"[Title/Abstract] OR "Computer-aided tongue"[Title/Abstract] OR "Tongue recognition"[Title/Abstract] OR "Tongue classification"[Title/Abstract] OR "Tongue segmentation"[Title/Abstract] OR "tongue photogra*"[Title/Abstract])

Embase (searched 23 February 2026)

'stroke'/exp OR 'cerebrovascular accident'/exp OR 'cerebral infarction'/exp OR 'brain ischemia'/exp OR 'brain hemorrhage'/exp OR (stroke* OR "cerebrovascular accident*" OR "cerebrovascular event*" OR CVA OR CVAs OR "cerebral infarction*" OR "brain infarction*" OR "cerebral stroke*" OR apoplexy OR "brain ischemia" OR "cerebral hemorrhage*" OR "intracranial hemorrhage*" OR "cerebrovascular disorder*"):ti,ab,kw)

AND

('tongue diagnosis'/exp OR 'traditional chinese medicine'/exp OR (tongue* OR glossoscop* OR "tongue image*" OR "tongue manifestation*" OR "tongue appearance*" OR "tongue coating*" OR "tongue fur*" OR "tongue body*" OR "tongue proper*" OR "digital tongue*" OR "automated tongue*" OR "computer-aided tongue*" OR "tongue recognition*" OR "tongue classification*" OR "tongue segmentation*" OR "tongue photogra*"):ti,ab,kw)

Web of Science Core Collection (searched 23 February 2026)

Advanced Search mode was used. The following topic terms were combined:

#1 TS=("stroke" OR "cerebrovascular accident" OR "cerebral infarction" OR "brain infarction" OR "brain ischemia" OR "cerebral hemorrhage" OR "apoplexy" OR "cerebrovascular disorders")

#2 TS=("tongue diagnosis" OR "glossoscopy" OR "tongue image*" OR "tongue manifestation" OR "tongue coating" OR "tongue body" OR "digital tongue" OR "automated tongue" OR "tongue recognition" OR "tongue classification")

#3 #1 AND #2

Results: 528 records.

CNKI (China National Knowledge Infrastructure; searched 23 February 2026)

Subject terms combined as follows:

主题：（中风＋中风后＋中风病＋中风患者＋中风后遗症＋中风偏瘫）OR（主题：中风＋中风急性期＋中风病人＋中风偏瘫患者＋中风恢复期＋中风后遗症患者）OR（主题：中风＋脑中风＋治疗脑中风＋急性中风＋混合性中风＋老年中风）OR（篇关摘：中风＋血瘀型中风＋气虚血瘀型中风，＋缺血中风＋类中风＋小中风（精确））AND（主题：舌诊+舌诊客观化＋舌诊研究＋舌诊客观化研究＋舌诊仪＋舌诊理论）OR（主题：舌诊＋舌诊图像＋舌诊方法＋舌诊信息＋舌诊辨证＋舌诊专家系统）OR（主题：舌诊＋中华舌诊观止+实用肝癌舌诊图谱+数字化舌诊＋计算机辅助舌诊＋数字舌诊）OR（主题：舌诊+计算机舌诊＋伤寒舌诊+计算机辅助舌诊系统）

Results: 6,215 records.

Wanfang Data (searched 23 February 2026)

主题:(中风 OR 脑卒中 OR 脑梗 OR 脑出血 OR 卒中 OR 脑血管意外) AND 主题:(舌 OR 舌诊 OR 舌象 OR 舌苔 OR 舌质 OR 望诊) AND (对照 OR 对比 OR 灵敏度 OR 特异度)

Results: 2,404 records.

VIP (Chongqing VIP; searched 23 February 2026)

Search 1: (M=中风 OR M=脑卒中 OR M=脑梗 OR M=脑出血) AND (M=舌诊 OR M=舌象 OR M=客观化 OR M=数字化)

Results: 200 records.

Search 2: (T=中风 OR T=脑卒中 OR T=脑梗) AND (T=舌诊 OR T=舌象 OR T=客观化)

Results: 127 records.

Combined VIP total: 327 records (after internal deduplication).

Supplementary Appendix S2: Studies excluded at full-text assessment

A total of 325 reports were excluded at the full-text assessment stage. The aggregate reasons for exclusion were: not a diagnostic accuracy study (n = 123), insufficient data for 2x2 contingency table construction (n = 66), incorrect reference standard (n = 57), unclear index test (n = 41), duplicate publications (n = 37), and overlapping patient cohort (n = 1). The overlapping cohort decision involving the Cao 2021 journal article and Cao 2021 master's thesis is described in Section 2.1 of the main manuscript.

**Report requiring clarification**	**Reason for exclusion**
Cao 2021 journal article	Overlapping cohort with Cao 2021 master's thesis; the more comprehensive thesis was retained to avoid unit-of-analysis error.

Supplementary Table S4: Leave-one-out sensitivity analysis results

Leave-one-out sensitivity analyses were performed for all six quantitatively pooled diagnostic clusters (k >= 3). All iterations used univariate random-effects models with the DerSimonian-Laird estimator, Freeman-Tukey double-arcsine transformation, and exact binomial study-level confidence intervals. For the Greasy Coating cluster (k = 4), the primary synthesis used a bivariate model, but leave-one-out iterations were reported as univariate descriptive analyses because excluding one study reduces the network to k = 3. The two Yin-Deficiency clusters (k = 2 each) were not eligible for leave-one-out analysis. These analyses are exploratory and should not be described as demonstrating stability when only two studies remain.

Cluster 1: Deficient-Cold Tongue (Pale tongue / White coating; k = 3)

Omitted study	Remaining k	Pooled Se (95% CI)	I² Se (%)	Pooled Sp (95% CI)	I² Sp (%)
None (baseline)	3	0.74 (0.36-0.99)	96.86	0.97 (0.87-1.00)	97.09
Cao 2021	2	0.74 (0.71–0.78)	N/A	0.96 (0.95–0.97)	N/A
Liu 2008	2	0.56 (0.51–0.61)	N/A	0.88 (0.85–0.90)	N/A
Xu 2025	2	0.93 (0.88–0.98)	N/A	0.99 (0.96–1.00)	N/A

Cluster 2: Qi-Deficiency Morphology (Fat / Teeth-marked tongue; k = 3)

Omitted study	Remaining k	Pooled Se (95% CI)	I² Se (%)	Pooled Sp (95% CI)	I² Sp (%)
None (baseline)	3	0.88 (0.73-0.98)	61.38	0.81 (0.38-1.00)	99.07
Cao 2021	2	0.91 (0.86–0.96)	N/A	0.93 (0.91–0.95)	N/A
Liu 2008	2	0.82 (0.70–0.93)	N/A	0.84 (0.81–0.87)	N/A
Yi 2024	2	0.92 (0.87–0.97)	N/A	0.96 (0.94–0.98)	N/A

Cluster 3: Blood Stasis Tongue (Purple tongue / Sublingual veins; k = 3)

Omitted study	Remaining k	Pooled Se (95% CI)	I² Se (%)	Pooled Sp (95% CI)	I² Sp (%)
None (baseline)	3	0.24 (0.07-0.46)	89.70	0.88 (0.60-1.00)	97.21
Zheng 2010	2	0.15 (0.11–0.19)	N/A	0.99 (0.98–1.00)	N/A
Gan 2018	2	0.26 (0.16–0.35)	N/A	0.76 (0.70–0.82)	N/A
Yi 2024	2	0.17 (0.13–0.22)	N/A	0.99 (0.98–1.00)	N/A

Cluster 4: Greasy Coating (Thick / Greasy coating; k = 4)

Omitted study	Remaining k	Pooled Se (95% CI)	I² Se (%)	Pooled Sp (95% CI)	I² Sp (%)
None (univariate baseline)	4	0.80 (0.61-0.94)	95.01	0.79 (0.70-0.87)	80.53
Cao 2021	3	0.80 (0.76–0.84)	N/A	0.75 (0.70–0.80)	40.09
Liu 2008	3	0.63 (0.56–0.69)	N/A	0.82 (0.68–0.95)	91.23
Gan 2018	3	0.77 (0.73–0.82)	N/A	0.82 (0.70–0.94)	92.20
Yi 2024	3	0.70 (0.53–0.87)	N/A	0.80 (0.63–0.96)	93.00

Cluster 5: Phlegm-Stasis Tongue (Dark-purple tongue / Sublingual veins; k = 3)

Omitted study	Remaining k	Pooled Se (95% CI)	I² Se (%)	Pooled Sp (95% CI)	I² Sp (%)
None (baseline)	3	0.78 (0.31-1.00)	98.17	0.79 (0.56-0.96)	94.27
Liu 2008	2	0.84 (0.80–0.88)	N/A	0.79 (0.72–0.86)	N/A
Yi 2024	2	0.94 (0.90–0.98)	N/A	0.90 (0.86–0.93)	N/A
Cao 2021	2	0.66 (0.60–0.72)	N/A	0.91 (0.88–0.94)	N/A

Cluster 6: Heat Syndrome Tongue (Red / Crimson tongue; k = 3)

Omitted study	Remaining k	Pooled Se (95% CI)	I² Se (%)	Pooled Sp (95% CI)	I² Sp (%)
None (baseline)	3	0.74 (0.50-0.93)	90.16	0.78 (0.55-0.95)	94.85
Cao 2021	2	0.63 (0.55–0.70)	N/A	0.87 (0.83–0.91)	N/A
Liu 2008	2	0.87 (0.81–0.94)	N/A	0.84 (0.79–0.89)	N/A
Zheng 2010	2	0.87 (0.81–0.92)	N/A	0.73 (0.68–0.77)	N/A

*N/A: I² not applicable when k = 2 (1 degree of freedom; between-study variance estimation unstable). * For LR/DOR calculations, a 0.5 continuity correction was applied only to studies with a zero cell. Sensitivity/specificity forest plots used original counts with Freeman-Tukey transformation and exact binomial study-level CIs. CI, confidence interval; Se, sensitivity; Sp, specificity.*

Supplementary Table S5: Full characteristics of included studies (corresponding to main Table 1)

This table provides the complete extraction fields abbreviated in main Table 1, including age/sex distribution, full syndrome categories, index-test procedures, AI-model notes, and reference-standard details. No study-level data were changed when moving these fields to the supplement.

Study (year)	Country	Design	Stroke type	Stage	N (case/ctrl)	Age, y	Sex, M/F	Target condition (TCM syndromes)	Index test	Reference standard
**Track 1: Manual visual tongue diagnosis (k = 6; N = 2,191)**										
Yi 2024a	China (Liaoning)	Cross-sectional, retrospective, single-center	Ischemic only	Recovery (2wk–6 mo)	170 (case-only)	64.1±8.4	97M/73F	(1) Wind-phlegm-stasis obstruction: 100/170 (58.8%); (2) Qi-deficiency with collateral stasis: 36/170 (21.2%); (3) Liver-kidney deficiency: 34/170 (20.0%)	Hybrid: manual eye-area inspection + smartphone tongue imaging (TCM App v1.2.0) with ML classification (6 algorithms)a	Chinese AIS Guidelines 2018; 14th Five-Year Plan TCM Internal Medicine Textbook (2021)
Zheng 2010	China (Hainan)	Descriptive, prospective, single-cohort, no controlb	Hemorrhagic only (ICH)c	Acute (≤24 h of admission)	88 (single-group)	58.5±13.2	61M/27F	Tongue body color (6 types) and coating (7 types) correlated with 6 TCM syndrome categories and severity	Manual visual tongue exam by ≥2 senior TCM physicians; standardized lighting; reference atlas	Guidelines for Prevention and Treatment of CVD in China (2005); National TCM Stroke Standards (1986)
Gan 2018	China (Fujian)	Retrospective cross-sectional	Ischemic only (ICD-10: I63)	Recovery (2 wk–6 mo)	420 (case-only)	65.3±14.2d	285M/135Fd	(1) Wind-phlegm obstructing collaterals: 248/420 (59.1%); (2) Qi-deficiency with blood stasis: 120/420 (28.6%); (3) Yin-deficiency with wind stirring: 52/420 (12.4%); (4) Phlegm-heat obstructing fu-organs: 0/420 (0%)	Manual TCM four-diagnosis; 6 tongue and pulse indices (thick coating, greasy coating, wiry pulse, slippery pulse, purplish tongue, hesitant pulse)	Guide to Diagnosis and Treatment of Common TCM Diseases (2008); 2017 SATCM Clinical Pathway
Liu 2008	China (Shandong)	Prospective cohort, serial assessment	Mixed: ischemic (n=229) + hemorrhagic (n=99)e	Acute (≤7 d)	328 (no control)	NRf	NRf	Tongue body and coating changes correlated with CT stroke subtype and 6 TCM syndrome groups	Manual visual tongue diagnosis by TCM physicians; serial observation from acute admission to recovery	CT for stroke subtype; national TCM guidelinesf
Xu 2025	China (Hunan)	Retrospective case-control, single-center	Acute ischemic only	Acute (onset ≤4 d)	1,035 (404 case/631 ctrl)g	Median (non-normal)h	Case: 203M/201F; Ctrl: 434M/197F	Qi-deficiency with blood stasis vs. other syndromes (syndrome-vs-syndrome design)g	Multi-domain TCM clinical assessment (tongue, pulse, symptoms); key tongue predictors: greasy coating (OR=3.58), dark tongue body (OR=1.78)	Chinese AIS Guidelines 2023; ZY/T001-2022

Cao 2021	China (Jiangsu)	Cross-sectional, prospective, single-center	Acute ischemic only	Acute (onset ≤14 d)	150 (case-only)	71.1±10.6	87M/63F	(1) Wind-phlegm-stasis obstruction: 108/150 (72.0%); (2) Wind-fire disturbing upper: 18/150 (12.0%); (3) Phlegm-heat obstructing fu-organs: 10/150 (6.7%); (4) Yin-deficiency with wind stirring: 9/150 (6.0%); (5) Qi-deficiency with blood stasis: 5/150 (3.3%)	Manual visual tongue exam by 3 senior TCM physicians (≥deputy-chief level); standardized digital photography (Panasonic DMC-ZS20); tongue body color (5 types), shape (5 types), coating (9 types), sublingual vessels	Chinese AIS Guidelines 2018; National TCM Stroke Diagnostic Standard (1996 trial edition)
**Track 2: AI-assisted tongue diagnosis (k = 4; N = 1,246) — qualitative synthesis; GRADE-DTA not applicable**										
Cui 2001	China (Beijing)	Prospective, cross-sectional, quantitative tongue-image analysis	Mixed: ischemic (n=256, 67.7%) + hemorrhagic (n=122, 32.3%)	All stages: acute 56.6%, recovery 25.9%, sequelae 17.5%	378 (single-group)	Ischemic: 60.3±10.2; Hemorrhagic: 58.5±9.8	252M/126F	(1) Wind-phlegm obstructing collaterals; (2) Phlegm-heat obstructing fu-organs; (3) Qi-deficiency with blood stasis; (4) Yin-deficiency with wind stirring; (5) Wind-fire disturbing upperi	Computerized RGB tongue color extraction (B/G, R/G, R+B/G ratios); no binary Se/Sp reportedi	ZY/T001.1-001 (1994/1996)
Wang 2023	China (Sichuan; Beijing)	AI model development; DenseNet201 + feature selection + Cubic SVM	Mixed: ischemic (n=182, 64.1%) + hemorrhagic (n=102, 35.9%)	Mixed acute + recovery (mean 56 d)	284 participants (852 images after augmentation)	Mean 64j	166M/118F	8 TCM syndromes (CACM ZYYXH/T 22-2008): 4 acute + 1 critical + 1 collapse + 2 recovery syndromes i	DenseNet201 CNN + 4-algorithm feature selection -> Cubic SVM; minimum syndrome-level accuracy 95.74%; best classifier accuracy 97.21%, F1 97.56% i	CACM Stroke Disease Standard ZYYXH/T 22-2008
Wang ZY 2024	China (Shandong)	Prospective healthy-control case-control, single-center	Acute ischemic only	Acute (onset <=2 wk)	300 (127 case/173 healthy controls)	Case: 66.32±10.29; Ctrl: 64.66±9.32	Case: 80M/47F; Ctrl: 90M/83F	Qi-deficiency with blood stasis in ACI vs. healthy volunteers i	ResNet18 and VGGNet16 CNNs; VGGNet16 best: Acc 90.67%, recall 85.60%, Sp 94.29%; no YOLOv8 reported in the primary thesis i	Chinese AIS Guidelines 2018; CACM Guidelines 2008
Han 2024	China (Shandong)	Prospective healthy-control case-control, single-center	Acute ischemic only	Acute (onset <=2 wk)	284 (111 case/173 healthy controls) k	Case: NR median; Ctrl: NR median l	Case: 61M/50F; Ctrl: 94M/79F	Liver-Qi stagnation in ACI vs. healthy volunteers i	ResNet18 + VGG16 CNNs; ResNet18 best: Acc 91.25%, recall 86.76%, Sp 94.13%; VGG16 Acc 88.21% i,m	GB/T16751.2-2021; Chinese AIS Guidelines 2018; CACM Guidelines 2008 m

Notes to Supplementary Table S5.

a Hybrid dual-modality design (manual eye-area inspection + tongue imaging via app). Placed in Track 1; ML models are exploratory secondary outcomes.
b Single-cohort descriptive study; all comparisons are intra-cohort subgroup analyses.
c ICH defined per the Guidelines for Prevention and Treatment of CVD in China (2005); exclusively hemorrhagic stroke patients enrolled.
d Data conflict between prior summary and primary publication. Table 1 uses primary published values (see Supplementary Material).
e Stroke subtype confirmed by English abstract (ischemic n=229, hemorrhagic n=99; total 328).
f Age and sex not reported in extractable form (OCR-unreadable scanned PDF). Age range 36–79 years visible in scanned image.
g Syndrome-versus-syndrome design; control group (n=631) comprises AIS patients with other syndromes, not healthy individuals. Rated high risk for spectrum bias in QUADAS-2.
h Age non-normally distributed; reported as median. Case group significantly older (P=0.003).

i Track 2 studies: none reports independent external validation. Binary accuracy metrics not reported in Cui 2001. GRADE-DTA not applicable; synthesis per SWiM guideline.
j Mean age 64 years (range 33–99); no SD reported.
k Data conflict (prior summary N=300 vs. primary thesis N=284). Primary thesis values used (see Supplementary Material).
l Group means only reported (case 66.25; control 64.53 years); non-normal distribution (P=0.156).
m Reference standard reported as GB/T16751.2-2021; potential overlap with the evaluated tongue features should be considered when interpreting model performance. The QUADAS-2 domain rating is reported as shown in Figure 2.
Abbreviations: Acc, accuracy; ACI, acute cerebral infarction; AIS, acute ischemic stroke; CACM, China Association of Chinese Medicine; CNN, convolutional neural network; CVD, cerebrovascular disease; F1, F1-score; ICH, intracerebral hemorrhage; ML, machine learning; NR, not reported; OR, odds ratio; Se, sensitivity; Sp, specificity; SVM, support vector machine; TCM, traditional Chinese medicine.

Supplementary Table S6: Complete 2x2 contingency tables and heterogeneity statistics

Eight diagnostic clusters organized under four TCM pathomechanisms.

1. Qi-Deficiency and Blood Stasis

Clinical feature cluster	k	Raw data per study (TP/FP/FN/TN)	Sens τ^2	Spec τ^2	Pooled Se (95% CI)	Pooled Sp (95% CI)	PLR (95% CI)	NLR (95% CI)	DOR (95% CI)
Deficient-Cold Tongue (Pale/White coating)	3	Cao: 3/0/2/145; Liu: 87/3/6/232; Xu: 227/76/177/555	0.38	0.12	0.74 (0.36, 0.99)	0.97 (0.87, 1.00)	31.0 (2.8, 341.6)	0.24 (0.07, 0.88)	146.7 (3.3, 6456.4)
Qi-Deficiency Morphology (Fat/Teeth-marked)	3	Cao: 3/8/2/137; Liu: 86/7/7/228; Yi: 30/87/6/47	0.05	0.64	0.88 (0.73, 0.98)	0.81 (0.38, 1.00)	7.4 (0.76, 71.7)	0.24 (0.07, 0.85)	30.2 (0.97, 940.6)
Blood Stasis Tongue (Purple/Sublingual)	3	Zheng: 18/8/18/44; Gan: 37/1/211/171; Yi: 5/39/31/95	0.14	0.32	0.24 (0.07, 0.46)	0.88 (0.60, 1.00)	2.83 (0.45, 17.68)	0.88 (0.65, 1.19)	3.58 (0.35, 36.50)

2. Wind-Phlegm and Dampness

Clinical feature cluster	k	Raw data per study (TP/FP/FN/TN)	Sens τ^2	Spec τ^2	Pooled Se (95% CI)	Pooled Sp (95% CI)	PLR (95% CI)	NLR (95% CI)	DOR (95% CI)
Greasy Coating (Thick/Greasy)	4	Cao: 59/2/49/40; Liu: 38/62/0/228; Gan: 206/47/42/125; Yi: 70/21/30/49	1.84	0.29	0.83 (0.52, 0.96)*	0.80 (0.68, 0.88)*	4.1 (2.5, 6.8)*	0.21 (0.06, 0.71)*	19.3 (4.6, 81.7)*
Phlegm-Stasis (Dark purple/Spots)	3	Cao: 102/21/6/21; Liu: 35/25/3/265; Yi: 35/9/65/61	0.70	0.19	0.78 (0.31, 1.00)	0.79 (0.56, 0.96)	3.8 (1.2, 12.7)	0.20 (0.04, 1.02)	18.8 (2.7, 132.1)

3. Phlegm-Heat and Excess Fire

Clinical feature cluster	k	Raw data per study (TP/FP/FN/TN)	Sens τ^2	Spec τ^2	Pooled Se (95% CI)	Pooled Sp (95% CI)	PLR (95% CI)	NLR (95% CI)	DOR (95% CI)
Heat Syndrome Tongue (Red/Crimson)	3	Cao: 27/56/1/66; Liu: 61/50/30/187; Zheng: 30/1/25/32	0.17	0.18	0.74 (0.50, 0.93)	0.78 (0.55, 0.95)	2.86 (1.76, 4.66)	0.42 (0.29, 0.59)	14.6 (4.6, 46.2)

4. Yin-Deficiency (Evidence Mapping)

Clinical feature cluster	k	Raw data per study (TP/FP/FN/TN)	Sens τ^2	Spec τ^2	Pooled Se (95% CI)	Pooled Sp (95% CI)	PLR (95% CI)	NLR (95% CI)	DOR (95% CI)
Yin-Deficiency Coating (Peeled/Scanty)	2	Cao: 6/1/3/140; Liu: 48/22/0/258	N/A*	N/A*	N/A*	N/A*	N/A*	N/A*	N/A*
Yin-Deficiency Body (Red/Thin)	2	Cao: 7/0/2/141; Liu: 29/0/19/280	N/A*	N/A*	N/A*	N/A*	N/A*	N/A*	N/A*

** N/A: Pooled estimates not calculated for k = 2 clusters to prevent spurious precision from unstable variance estimation. Individual study estimates are presented as unpooled evidence maps in Figure 6. † Blood Stasis Tongue estimates were recalculated from the verified 2x2 tables; the cluster showed low sensitivity and imprecise likelihood ratios rather than a reliable inverse diagnostic association. TP, true positive; FP, false positive; FN, false negative; TN, true negative; Se, sensitivity; Sp, specificity; PLR, positive likelihood ratio; NLR, negative likelihood ratio; DOR, diagnostic odds ratio; τ², between-study variance; CI, confidence interval.*

Supplementary Table S7: Detailed STARD-AI/TRIPOD+AI-informed audit of AI-assisted tongue diagnosis studies. This table summarizes model-level methodology, data structure and image-to-participant ratio, image-acquisition details, preprocessing and augmentation, validation design, patient-level split reporting, baseline/human-comparator benchmarking, calibration, decision-curve analysis, external validation, comparator type, and applicability/leakage concerns. NR indicates not reported in the source publication; N/A indicates not applicable to the study design or model output.

Model architecture / feature approach	Data structure and image-to-participant ratio	Image-acquisition device or protocol	Preprocessing	Augmentation	Training/test split	Patient-level split reported	Independent external validation reported	Calibration reported	Decision-curve analysis reported	Baseline / human expert comparator reported	Comparator type / diagnostic task	Principal leakage, circularity, and applicability concern
Cui et al. (2001): RGB-based expert system / quantitative color-feature analysis	378 stroke patients; number of tongue images not separately reported; image-to-participant ratio not estimable.	OLYMPUS digital camera; photographs input into computer and processed using a TCM tongue-diagnosis expert system. Additional lighting/white-balance protocol NR.	Tongue-image segmentation/processing by the expert system; RGB color values and B/G, R/G, and R+B/G ratio features extracted.	N/A/NR	N/A/NR; group-level quantitative analysis only.	N/A/NR	NR	NR	NR	No simple baseline model or model-vs-independent expert TCM physician comparator reported.	Stroke patients with different TCM syndrome groups; no healthy-control comparator.	No prespecified diagnostic threshold or individual-level classification model reported; early group-level quantitative color analysis; limited acquisition reporting; independence of syndrome labels from tongue features not demonstrated.
Wang et al. (2023): ImageNet-pretrained DenseNet201 feature extraction + information gain/chi-square/symmetric uncertainty/ReliefF feature selection + Cubic SVM	284 stroke patients; 284 original tongue images increased to 852 images after augmentation (approximately 3 images per participant after augmentation).	Tongue images from 284 stroke patients; acquisition hardware, lighting, and white-balance protocol NR.	Image cropping/adaptation to DenseNet201 input; DenseNet201 transfer-learning feature extraction; feature-vector selection using IG/chi-square/SU/ReliefF.	Random rotation (40%), horizontal flip (20%), random crop (10%), random scaling (10%), random shift (10%), Gaussian noise (50%); images increased from 284 to 852.	Internal 3-fold and 6-fold cross-validation; no independent external test set reported.	NR; patient-level separation for augmented images not reported.	NR	NR	NR	No simple baseline model or model-vs-independent expert TCM physician comparator reported.	Eight-class TCM stroke syndrome classification among stroke patients.	Promising reported internal multiclass performance, but acquisition conditions were incompletely reported. If feature selection or augmentation was performed before resampling, or augmented images were not kept within patient-level folds, internal performance may be optimistic; no independent external validation or calibration reported.
Wang ZY et al. (2024): ImageNet-pretrained ResNet18 and VGGNet16 CNNs; global average pooling, dense layer, Dropout 0.2, binary cross-entropy loss	300 participants; 3,000 images; 10 images per participant.	BOSMA G1 camera (7680 x 4320, 8K UHD) + OLYMPUS M.ZUIKO DIGITAL ED 40-150 mm F2.8 PRO lens; ISO <=400; gray-card color-temperature measurement/manual white balance; autofocus; tripod/safe shutter; 6500K/D65-like lighting, Ra>90, E<=32 lx, neutral-gray environment.	Poor-quality images excluded; cropping/interference removal; JPG storage; resize to 224 x 224; ToTensor and Normalize using ImageNet mean/std.	RandomHorizontalFlip and RandomRotation(10).	Image-level 80/20 split: 2,400 training images and 600 testing images from 3,000 images.	NR; image-level split reported, but patient-level split not reported.	NR	NR	NR	No simple baseline model or model-vs-independent expert TCM physician comparator reported.	Acute ischemic stroke with Qi-deficiency and Blood-stasis syndrome vs healthy controls.	Healthy-control case-control comparator creates spectrum/applicability concerns and may overestimate performance for real-world syndrome differentiation among stroke patients; possible patient-level leakage/non-independent image-level split because multiple images per participant were split at image level and patient-level separation was not reported; no independent external validation or calibration reported.
Han et al. (2024): ImageNet-pretrained ResNet18 and VGG16 CNNs; global average pooling, dense layer, Dropout 0.2, binary cross-entropy loss	284 participants; 2,840 images; 10 images per participant.	ISO <=400; gray-card color-temperature measurement/manual white balance; autofocus; tripod/safe shutter; 6500K/D65-like lighting, Ra>90, E<=32 lx, neutral-gray environment; exact camera/lens model NR.	Poor-quality images excluded; rotation correction/cropping; resize to 224 x 224; ToTensor and Normalize using ImageNet mean/std.	RandomHorizontalFlip and RandomRotation(10).	Image-level 80/20 split: 2,280 training images and 560 testing images from 2,840 images.	NR; image-level split reported, but patient-level split not reported.	NR	NR	NR	No simple baseline model or model-vs-independent expert TCM physician comparator reported.	Acute cerebral infarction with Liver-Qi stagnation syndrome vs healthy controls.	Healthy-control case-control comparator creates spectrum/applicability concerns and may overestimate performance for real-world syndrome differentiation among stroke patients; possible patient-level leakage/non-independent image-level split because multiple images per participant were split at image level and patient-level separation was not reported; no independent external validation or calibration reported.

Supplementary Table S8: Three-scenario pre-test probability analyses (20%, 50%, 80%)

Post-test probabilities were calculated using Bayes' theorem: post-test odds = pre-test odds x likelihood ratio; post-test probability = post-test odds / (1 + post-test odds). PLR and NLR values were taken from the verified pooled LR analyses in Supplementary Table S6; for Greasy Coating, the primary bivariate LR values were used.

Cluster	PLR	NLR	Post-test prob (PLR+) at 20%	Post-test prob (PLR+) at 50%	Post-test prob (PLR+) at 80%	Post-test prob (NLR−) at 20%	Post-test prob (NLR−) at 50%	Post-test prob (NLR−) at 80%
Deficient-Cold Tongue	31.05	0.24	88.6%	96.9%	99.2%	5.7%	19.6%	49.4%
Qi-Deficiency Morphology	7.4	0.24	64.9%	88.1%	96.7%	5.7%	19.5%	49.3%
Blood Stasis Tongue†	2.83	0.88	41.5%	73.9%	91.9%	18.0%	46.8%	77.9%
Greasy Coating	4.11	0.21	50.7%	80.4%	94.3%	5.1%	17.6%	46.0%
Phlegm-Stasis Tongue	3.82	0.2	48.8%	79.2%	93.9%	4.9%	16.9%	44.9%
Heat Syndrome Tongue	2.86	0.42	41.7%	74.1%	92.0%	9.4%	29.4%	62.5%

† Blood Stasis Tongue estimates are imprecise and should not be interpreted as showing inverse diagnostic performance. PLR, positive likelihood ratio; NLR, negative likelihood ratio.

Supplementary Table S9. 2x2 data provenance, back-calculation, and rounding-residual audit

This table documents how each TP/FP/FN/TN table used in the diagnostic meta-analysis was obtained from the original reports. It separates directly reported or directly extracted counts from back-calculated or derived rows and documents denominator checks and rounding residuals. No unreported values were imputed.

Panel A. Row-level source and rounding-residual audit

Feature cluster	Study	Target condition / denominator	Index tongue feature	TP	FP	FN	TN	Source type	Basis in original report	Rounding / residual audit
Deficient-Cold Tongue	Cao 2021	Qi-deficiency with blood-stasis (5) vs others (145)	Pale tongue	3	0	2	145	Direct cross-tab	Cao 2021 Table 10: tongue color by five TCM syndrome types	TP+FN=5; FP+TN=145; exact counts
Qi-Deficiency Morphology	Cao 2021	Qi-deficiency with blood-stasis (5) vs others (145)	Puffy/enlarged tongue	3	8	2	137	Direct cross-tab	Cao 2021 Table 11: tongue shape by five TCM syndrome types	TP+FN=5; FP+TN=145; exact counts
Greasy/Coating-related cluster	Cao 2021	Wind-phlegm-stasis obstruction (108) vs others (42)	White greasy coating	59	2	49	40	Direct cross-tab	Cao 2021 Table 13: tongue coating by five TCM syndrome types	TP+FN=108; FP+TN=42; exact counts
Phlegm-Stasis Tongue	Cao 2021	Wind-phlegm-stasis obstruction (108) vs others (42)	Sublingual petechiae/ecchymosis	102	21	6	21	Direct cross-tab	Cao 2021 Table 14: sublingual collateral findings by five TCM syndrome types	TP+FN=108; FP+TN=42; exact counts
Heat Syndrome Tongue	Cao 2021	Heat-related syndromes (28) vs others (122)	Red tongue	27	56	1	66	Direct grouped cross-tab	Cao 2021 Table 10: tongue color by five TCM syndrome types	TP+FN=28; FP+TN=122; exact counts
Yin-Deficiency Coating	Cao 2021	Yin-deficiency with wind stirring (9) vs others (141)	Scant white coating	6	1	3	140	Direct cross-tab	Cao 2021 Table 13: tongue coating by five TCM syndrome types	TP+FN=9; FP+TN=141; exact counts
Yin-Deficiency Body	Cao 2021	Yin-deficiency with wind stirring (9) vs others (141)	Thin tongue body	7	0	2	141	Direct cross-tab	Cao 2021 Table 11: tongue shape by five TCM syndrome types	TP+FN=9; FP+TN=141; exact counts
Deficient-Cold Tongue	Liu 2008	Qi-deficiency collateral obstruction (93) vs others (235)	Pale tongue	87	3	6	232	Direct cross-tab	Liu 2008 Table 2: tongue features by TCM syndrome type	TP+FN=93; FP+TN=235; exact counts
Qi-Deficiency Morphology	Liu 2008	Qi-deficiency collateral obstruction (93) vs others (235)	Puffy/enlarged tongue	86	7	7	228	Direct cross-tab	Liu 2008 Table 2: tongue features by TCM syndrome type	TP+FN=93; FP+TN=235; exact counts
Greasy/Coating-related cluster	Liu 2008	Phlegm-confusing mind (38) vs others (290)	White greasy coating	38	62	0	228	Direct cross-tab	Liu 2008 Table 2: tongue features by TCM syndrome type	TP+FN=38; FP+TN=290; exact counts; zero FN cell
Phlegm-Stasis Tongue	Liu 2008	Phlegm-confusing mind (38) vs others (290)	Purple-dark tongue	35	25	3	265	Direct cross-tab	Liu 2008 Table 2: tongue features by TCM syndrome type	TP+FN=38; FP+TN=290; exact counts
Heat Syndrome Tongue	Liu 2008	Phlegm-heat exuberance (91) vs others (237)	Crimson/red tongue	61	50	30	187	Direct cross-tab	Liu 2008 Table 2: tongue features by TCM syndrome type	TP+FN=91; FP+TN=237; exact counts
Yin-Deficiency Coating	Liu 2008	Yin-deficiency with yang hyperactivity (48) vs others (280)	Scant or peeled coating	48	22	0	258	Direct cross-tab	Liu 2008 Table 2: tongue features by TCM syndrome type	TP+FN=48; FP+TN=280; exact counts; zero FN cell
Yin-Deficiency Body	Liu 2008	Yin-deficiency with yang hyperactivity (48) vs others (280)	Red tongue	29	0	19	280	Direct cross-tab	Liu 2008 Table 2: tongue features by TCM syndrome type	TP+FN=48; FP+TN=280; exact counts; zero FP cell
Greasy/Coating-related cluster	Gan 2018	Phlegm-stasis obstructing channels	Greasy fur/coating; variable 2	206	47	42	125	Back-calculated from Se/Sp	Gan 2018 Tables 4, 7 and 8: syndrome denominator, candidate variable	round(0.8306*248)=206; round(0.7267*172)=125; residual

Feature cluster	Study	Target condition / denominator	Index tongue feature	TP	FP	FN	TN	Source type	Basis in original report	Rounding / residual audit
		(248) vs others (172)							definition, and diagnostic metrics	consistent with percentage rounding
Blood Stasis Tongue	Gan 2018	Phlegm-stasis obstructing channels (248) vs others (172)	Purplish tongue; variable 5	37	1	211	171	Back-calculated from Se/Sp	Gan 2018 Tables 4, 7 and 8: syndrome denominator, candidate variable definition, and diagnostic metrics	round(0.1492*248)=37; round(0.9942*172)=171; residual consistent with percentage rounding
Deficient-Cold Tongue	Xu 2025	AIS with Qi-deficiency and blood-stasis (404) vs AIS controls (631)	White tongue coating	227	76	177	555	Direct two-group counts	Xu 2025 Figure 1 and Table 2: case/control denominators and symptoms/signs comparison	TP+FN=404; FP+TN=631; exact counts
Blood Stasis Tongue	Zheng 2010	Blood-stasis syndrome (36) vs patients without blood-stasis syndrome (52)	Purple tongue	18	8	18	44	Derived from overlapping labels	Zheng 2010 Tables 1 and 2: total purple tongues and blood-stasis syndrome by tongue color	Total purple tongues=26; blood-stasis purple=18; FP=26-18=8; TN=88-36-8=44
Heat Syndrome Tongue	Zheng 2010	Fire-heat syndrome (55) vs patients without fire-heat syndrome (33)	Red tongue	30	1	25	32	Derived from overlapping labels	Zheng 2010 Tables 1 and 2: total red tongues and fire-heat syndrome by tongue color	Total red tongues=31; fire-heat red=30; FP=31-30=1; TN=88-55-1=32
Qi-Deficiency Morphology	Yi 2024	Qi-deficiency collateral-stasis (36) vs other syndrome groups (134)	Tooth-marked tongue	30	87	6	47	Direct cross-tab	Yi 2024 three-syndrome tongue-feature summary table: tongue shape and sublingual-collateral classification by syndrome	TP+FN=36; FP+TN=134; exact counts
Blood Stasis Tongue	Yi 2024	Qi-deficiency collateral-stasis (36) vs other syndrome groups (134)	Purple sublingual-collateral color	5	39	31	95	Direct cross-tab	Yi 2024 three-syndrome tongue-feature summary table: sublingual-collateral color by syndrome	TP+FN=36; FP+TN=134; exact counts
Greasy/Coating-related cluster	Yi 2024	Wind-phlegm-stasis obstruction (100) vs non-wind-phlegm-stasis (70)	Thick coating/coating-thickness feature	70	21	30	49	Direct cross-tab	Yi 2024 two-syndrome tongue-feature summary table (Table 33): coating thickness by wind-phlegm-stasis vs non-wind-phlegm-stasis	TP+FN=100; FP+TN=70; exact counts; note feature is thick coating
Phlegm-Stasis Tongue	Yi 2024	Wind-phlegm-stasis obstruction (100) vs non-wind-phlegm-stasis (70)	Purple sublingual-collateral color	35	9	65	61	Direct cross-tab	Yi 2024 two-syndrome tongue-feature summary table (Table 33): sublingual-collateral color by wind-phlegm-stasis vs non-wind-phlegm-stasis	TP+FN=100; FP+TN=70; exact counts

Supplementary Table S10. Methodological sensitivity analyses and model-diagnostics audit

This table summarizes model-diagnostic and methodological sensitivity analyses for sparse diagnostic meta-analysis estimates. The 2x2 provenance, back-calculation, and rounding-residual audit defining affected rows is provided separately in Supplementary Table S9. Estimates are shown as point estimate (95% CI) where applicable. Primary meta-analysis was restricted to clusters with k >= 3; k = 2 estimates after exclusion were calculated only as descriptive influence checks and were not interpreted as confirmatory pooled estimates.

Panel A. Greasy Coating bivariate and GLMM model diagnostics

Model	Data	Converged	Quantity	Estimate	95% CI	Interpretation / caution
Bivariate Reitsma model (metandi)	All Greasy Coating rows (k=4)	Yes	Log likelihood	-32.071697	Not applicable	Primary bivariate SROC model; convergence was achieved.
Bivariate Reitsma model (metandi)	All Greasy Coating rows (k=4)	Yes	Se	0.83	0.52-0.96	Boundary-respecting metandi CI retained for primary inference.
Bivariate Reitsma model (metandi)	All Greasy Coating rows (k=4)	Yes	Sp	0.80	0.68-0.88	Boundary-respecting metandi CI retained for primary inference.
Bivariate Reitsma model (metandi)	All Greasy Coating rows (k=4)	Yes	LR+	4.11	2.50-6.77	Primary bivariate summary estimate; interpreted cautiously because the model included only four studies.
Bivariate Reitsma model (metandi)	All Greasy Coating rows (k=4)	Yes	LR-	0.21	0.06-0.71	Primary bivariate summary estimate; interpreted cautiously because the model included only four studies.
Bivariate Reitsma model (metandi)	All Greasy Coating rows (k=4)	Yes	DOR	19.31	4.57-81.66	Primary bivariate summary estimate; interpreted cautiously because the model included only four studies.
Bivariate Reitsma model (metandi)	All Greasy Coating rows (k=4)	Yes	Var(logitSe)	1.842	0.209-16.226	Random-effects parameter from the primary bivariate model; interpreted cautiously because the model included only four studies.
Bivariate Reitsma model (metandi)	All Greasy Coating rows (k=4)	Yes	Var(logitSp)	0.294	0.022-3.929	Random-effects parameter from the primary bivariate model; interpreted cautiously because the model included only four studies.
Bivariate Reitsma model (metandi)	All Greasy Coating rows (k=4)	Yes	Corr(logits)	-0.358	-0.904-0.633	Random-effects parameter from the primary bivariate model; interpreted cautiously because the model included only four studies.
Bivariate Reitsma model (metandi)	All Greasy Coating rows (k=4)	Yes	Derived random-effects covariance matrix	[[1.842, -0.263], [-0.263, 0.294]]	Not applicable	Derived from Var(logitSe), Var(logitSp), and Corr(logits); distinct from the covariance between fixed-effect estimates reported by Stata.
Unstructured binomial GLMM	All Greasy Coating rows (k=4)	Yes	Log likelihood	-32.070337	Not applicable	Sensitivity model structurally close to the bivariate model.
Unstructured binomial GLMM	All Greasy Coating rows (k=4)	Yes	Se	0.83	Not used for inference	Probability-scale delta CI exceeded 1; metandi CI was retained as the primary boundary-respecting interval.
Unstructured binomial GLMM	All Greasy Coating rows (k=4)	Yes	Sp	0.80	0.70-0.90	Probability-scale margin; interpreted only as a model-consistency check.
Reduced random-intercept GLMM	All Greasy Coating rows (k=4)	Yes	Se / Sp	0.76 / 0.74	Se 0.69-0.82; Sp 0.67-0.81	Exploratory reduced model; not structurally equivalent to Reitsma.
Bivariate Reitsma	Greasy Coating rows	No	Refit status	Not fitted	Not applicable	Stata metandi returned code 2001: metandi requires at least

Model	Data	Converged	Quantity	Estimate	95% CI	Interpretation / caution
model after excluding Gan2018	excluding Gan2018 (k=3)					four studies; univariate FT/logit exclusion sensitivity was therefore used.

Panel B. Freeman-Tukey primary estimates versus logit-transformed sensitivity analysis

Cluster	Metric	k	Primary Freeman-Tukey	Logit sensitivity	Absolute difference	Interpretation
Deficient-Cold Tongue	Sensitivity	3	0.74 (0.36-0.99)	0.76 (0.34-0.95)	0.02	Broadly similar point estimate; CI remained wide where heterogeneity was high
Deficient-Cold Tongue	Specificity	3	0.97 (0.87-1.00)	0.98 (0.83-1.00)	0.01	Broadly similar point estimate; CI remained wide where heterogeneity was high; 0.5 correction for boundary cell
Qi-Deficiency Morphology	Sensitivity	3	0.88 (0.73-0.98)	0.85 (0.68-0.94)	0.03	Broadly similar point estimate; CI remained wide where heterogeneity was high
Qi-Deficiency Morphology	Specificity	3	0.81 (0.38-1.00)	0.87 (0.27-0.99)	0.06	Broadly similar point estimate; CI remained wide where heterogeneity was high
Blood Stasis Tongue	Sensitivity	3	0.24 (0.07-0.46)	0.23 (0.09-0.50)	0.01	Broadly similar point estimate; CI remained wide where heterogeneity was high
Blood Stasis Tongue	Specificity	3	0.88 (0.60-1.00)	0.90 (0.67-0.98)	0.02	Broadly similar point estimate; CI remained wide where heterogeneity was high
Greasy Coating	Sensitivity	4	0.80 (0.61-0.94)	0.76 (0.57-0.88)	0.04	Broadly similar point estimate; CI remained wide where heterogeneity was high; 0.5 correction for boundary cell
Greasy Coating	Specificity	4	0.79 (0.70-0.87)	0.77 (0.69-0.84)	0.02	Broadly similar point estimate; CI remained wide where heterogeneity was high
Phlegm-Stasis Tongue	Sensitivity	3	0.78 (0.31-1.00)	0.82 (0.26-0.98)	0.04	Broadly similar point estimate; CI remained wide where heterogeneity was high
Phlegm-Stasis Tongue	Specificity	3	0.79 (0.56-0.96)	0.81 (0.49-0.95)	0.02	Broadly similar point estimate; CI remained wide where heterogeneity was high
Heat Syndrome Tongue	Sensitivity	3	0.74 (0.50-0.93)	0.70 (0.49-0.85)	0.04	Broadly similar point estimate; CI remained wide where heterogeneity was high
Heat Syndrome Tongue	Specificity	3	0.78 (0.55-0.95)	0.78 (0.53-0.91)	0.0	Broadly similar point estimate; CI remained wide where heterogeneity was high

Panel C. Affected-row exclusion sensitivity under primary and alternative transformation models

Scenario	Cluster	Metric	Pooling status	Freeman-Tukey estimate	Change from all-study FT estimate	Logit estimate	Interpretation
Primary affected-row exclusion (Gan2018 and	Blood Stasis Tongue	Sensitivity	Not pooled	Not pooled	Not estimable	Not pooled	Only Yi2024 remained; pooled estimate not interpretable

Scenario	Cluster	Metric	Pooling status	Freeman-Tukey estimate	Change from all-study FT estimate	Logit estimate	Interpretation
Zheng2010)							
Primary affected-row exclusion (Gan2018 and Zheng2010)	Blood Stasis Tongue	Specificity	Not pooled	Not pooled	Not estimable	Not pooled	Only Yi2024 remained; pooled estimate not interpretable
Primary affected-row exclusion (Gan2018 and Zheng2010)	Deficient-Cold Tongue	Sensitivity	k=3	0.74 (0.36-0.99)	0.00	0.76 (0.34-0.95)	Point estimate remained broadly comparable; interpretation limited by high heterogeneity
Primary affected-row exclusion (Gan2018 and Zheng2010)	Deficient-Cold Tongue	Specificity	k=3	0.97 (0.87-1.00)	0.00	0.98 (0.83-1.00)	Point estimate remained broadly comparable; interpretation limited by high heterogeneity
Primary affected-row exclusion (Gan2018 and Zheng2010)	Greasy Coating	Sensitivity	k=3	0.80 (0.49-0.98)	0.00	0.70 (0.48-0.85)	Point estimate remained broadly comparable; interpretation limited by high heterogeneity
Primary affected-row exclusion (Gan2018 and Zheng2010)	Greasy Coating	Specificity	k=3	0.82 (0.69-0.92)	+0.03	0.80 (0.67-0.89)	Point estimate remained broadly comparable; interpretation limited by high heterogeneity
Primary affected-row exclusion (Gan2018 and Zheng2010)	Heat Syndrome Tongue	Sensitivity	k=2	0.76 (0.67-0.83)	+0.02	0.86 (0.33-0.99)	Reduced to k=2; descriptive influence check only
Primary affected-row exclusion (Gan2018 and Zheng2010)	Heat Syndrome Tongue	Specificity	k=2	0.71 (0.66-0.76)	-0.07	0.68 (0.40-0.87)	Reduced to k=2; descriptive influence check only
Primary affected-row exclusion (Gan2018 and Zheng2010)	Phlegm-Stasis Tongue	Sensitivity	k=3	0.78 (0.31-1.00)	0.00	0.82 (0.26-0.98)	Point estimate remained broadly comparable; interpretation limited by high heterogeneity
Primary affected-row exclusion (Gan2018 and Zheng2010)	Phlegm-Stasis Tongue	Specificity	k=3	0.79 (0.56-0.96)	0.00	0.81 (0.49-0.95)	Point estimate remained broadly comparable; interpretation limited by high heterogeneity
Primary affected-row exclusion (Gan2018 and Zheng2010)	Qi-Deficiency Morphology	Sensitivity	k=3	0.88 (0.73-0.98)	0.00	0.85 (0.68-0.94)	Point estimate remained broadly comparable; interpretation limited by high heterogeneity
Primary affected-row exclusion (Gan2018 and Zheng2010)	Qi-Deficiency Morphology	Specificity	k=3	0.81 (0.38-1.00)	0.00	0.87 (0.27-0.99)	Point estimate remained broadly comparable; interpretation limited by high heterogeneity
Additional conservative influence exclusion (also excluding Xu2025)	Blood Stasis Tongue	Sensitivity	Not pooled	Not pooled	Not estimable	Not pooled	Only Yi2024 remained; pooled estimate not interpretable
Additional conservative influence exclusion (also excluding Xu2025)	Blood Stasis Tongue	Specificity	Not pooled	Not pooled	Not estimable	Not pooled	Only Yi2024 remained; pooled estimate not interpretable
Additional conservative influence exclusion (also excluding Xu2025)	Deficient-Cold Tongue	Sensitivity	k=2	0.96 (0.89-1.00)	+0.22	0.84 (0.37-0.98)	Reduced to k=2 after Xu2025 exclusion; descriptive influence check only

Scenario	Cluster	Metric	Pooling status	Freeman-Tukey estimate	Change from all-study FT estimate	Logit estimate	Interpretation
Additional conservative influence exclusion (also excluding Xu2025)	Deficient-Cold Tongue	Specificity	k=2	0.99 (0.98-1.00)	+0.02	0.99 (0.97-1.00)	Reduced to k=2 after Xu2025 exclusion; descriptive influence check only
Additional conservative influence exclusion (also excluding Xu2025)	Greasy Coating	Sensitivity	k=3	0.80 (0.49-0.98)	0.00	0.70 (0.48-0.85)	Point estimate remained broadly comparable; interpretation limited by high heterogeneity
Additional conservative influence exclusion (also excluding Xu2025)	Greasy Coating	Specificity	k=3	0.82 (0.69-0.92)	+0.03	0.80 (0.67-0.89)	Point estimate remained broadly comparable; interpretation limited by high heterogeneity
Additional conservative influence exclusion (also excluding Xu2025)	Heat Syndrome Tongue	Sensitivity	k=2	0.76 (0.67-0.83)	+0.02	0.86 (0.33-0.99)	Reduced to k=2; descriptive influence check only
Additional conservative influence exclusion (also excluding Xu2025)	Heat Syndrome Tongue	Specificity	k=2	0.71 (0.66-0.76)	-0.07	0.68 (0.40-0.87)	Reduced to k=2; descriptive influence check only
Additional conservative influence exclusion (also excluding Xu2025)	Phlegm-Stasis Tongue	Sensitivity	k=3	0.78 (0.31-1.00)	0.00	0.82 (0.26-0.98)	Point estimate remained broadly comparable; interpretation limited by high heterogeneity
Additional conservative influence exclusion (also excluding Xu2025)	Phlegm-Stasis Tongue	Specificity	k=3	0.79 (0.56-0.96)	0.00	0.81 (0.49-0.95)	Point estimate remained broadly comparable; interpretation limited by high heterogeneity
Additional conservative influence exclusion (also excluding Xu2025)	Qi-Deficiency Morphology	Sensitivity	k=3	0.88 (0.73-0.98)	0.00	0.85 (0.68-0.94)	Point estimate remained broadly comparable; interpretation limited by high heterogeneity
Additional conservative influence exclusion (also excluding Xu2025)	Qi-Deficiency Morphology	Specificity	k=3	0.81 (0.38-1.00)	0.00	0.87 (0.27-0.99)	Point estimate remained broadly comparable; interpretation limited by high heterogeneity
